# Integrative Approach to Risk Factors in Simple Chronic Obstructive Airway Diseases of the Lung or Associated with Metabolic Syndrome—Analysis and Prediction

**DOI:** 10.3390/nu16121851

**Published:** 2024-06-13

**Authors:** Liliana Streba, Violeta Popovici, Andreea Mihai, Magdalena Mititelu, Carmen Elena Lupu, Marius Matei, Ionela Mihaela Vladu, Maria Livia Iovănescu, Ramona Cioboată, Cristina Călărașu, Ștefan Sebastian Busnatu, Costin-Teodor Streba

**Affiliations:** 1 Department of Oncology, University of Medicine and Pharmacy of Craiova, 200349 Craiova, Romania; liliana.streba@umfcv.ro; 2 Center for Mountain Economics, “Costin C. Kiriţescu” National Institute of Economic Research (INCE-CEMONT), Romanian Academy, 725700 Vatra-Dornei, Romania; violeta.popovici@ce-mont.ro; 3 Department of Pulmonology, University of Medicine and Pharmacy of Craiova, 200349 Craiova, Romania; ramona.cioboata@umfcv.ro (R.C.); cristina.calarasu@umfcv.ro (C.C.); costin.streba@umfcv.ro (C.-T.S.); 4 Faculty of Pharmacy, “Carol Davila” University of Medicine and Pharmacy, 020956 Bucharest, Romania; 5 Department of Mathematics and Informatics, Faculty of Pharmacy, “Ovidius” University of Constanta, 900001 Constanta, Romania; clupu@univ-ovidius.ro; 6 Department of Histology, University of Medicine and Pharmacy of Craiova, 200349 Craiova, Romania; marius.matei@umfcv.ro; 7 Department of Diabetes, Nutrition and Metabolic Diseases, University of Medicine and Pharmacy of Craiova, 200349 Craiova, Romania; ionela.vladu@umfcv.ro; 8 Department of Cardiology, University of Medicine and Pharmacy of Craiova, 200349 Craiova, Romania; maria.iovanescu@umfcv.ro; 9 Department of Cardio-Thoracic Pathology, Faculty of Medicine, “Carol Davila” University of Medicine and Pharmacy, 050474 Bucharest, Romania; stefan.busnatu@umfcd.ro

**Keywords:** diabetes mellitus, respiratory dysfunctions, behavioral risk factors, dietary habits, lifestyle choices

## Abstract

We conducted an epidemiological non-interventional cross-sectional and case-control study from 1 January 2023 until 26 May 2023 in Oltenia region, southwestern Romania. Throughout the research, 160 consecutive patients were included from two different clinical departments (1—Pneumology; 2—Diabetes and Nutritional Diseases). Subjects were voluntary adult individuals of any gender who expressed their written consent. The clinical data of the patients were correlated with the exposure to behavioral risk factors (diet, lifestyle, exposure to pollutants) to identify some negative implications that could be corrected to improve the quality of life of patients with simple chronic obstructive airway diseases of the lung or associated with metabolic syndrome (MS). In the first group of patients with respiratory diseases, there was a higher degree of exposure to toxic substances (43.75%) compared to the second group of patients with diabetes (18.75%); it is also noticeable that in the first group, there were noticeably fewer individuals who have never smoked (25%) compared to the second group (50%). Respiratory function impairment was observed to be more severe in overweight individuals. In the group of patients with known lung diseases, a positive correlation was noted between the presence of MS and respiratory dysfunctions of greater severity. Additionally, potential exacerbating factors affecting lung function, such as direct exposure to toxins and smoking, were considered. Potential secondary factors exacerbating respiratory dysfunction were considered by correlating biochemical parameters with dietary habits. These included reduced consumption of vegetables, inadequate hydration, and increased intake of sweets and products high in saturated or trans fats (commonly found in junk food), primarily due to their potential contribution to excess weight. Compared to patients without MS, the severity of the pulmonary function impairment correlated with the number of criteria met for MS and, independently, with an increase in weight.

## 1. Introduction

Many conditions exist within the broad spectrum of bronchopulmonary pathologies, each with varying progression potential and significant implications for morbidity and overall mortality. Among chronic obstructive respiratory diseases, bronchial asthma and, notably, chronic obstructive pulmonary disease (COPD) stand out. The latter condition currently ranks as the third most common cause of death in the general population [1,2,3].

Each of the mentioned pathological entities presents distinctive clinical and evolutive features, including distinct clinical phenotypes as observed in bronchial asthma and multiple stages of progression within the context of COPD [4,5].

Asthma is widely acknowledged to considerably impact the global population, with an estimated 262 million individuals affected by this condition in 2019 [6]. In contrast, COPD, with its significantly higher prevalence, is regarded as one of the most prominent conditions in the field of pulmonary pathology. This distinction is not only attributable to its elevated incidence but also, more importantly, from the implications driven by its progressive nature and the ominous outcome represented by the possibility of developing chronic respiratory failure or chronic cor pulmonale, a pathological state that, at a certain point, might become refractory to therapeutic interventions, either partially or entirely [3,6,7,8,9].

In the current landscape of general pathology, a distinct position is occupied by metabolic syndrome (MS), a complex entity encompassing multiple elements, each representing genuine pathological conditions. The combination of these components yields profoundly adverse implications for the overall health status of individuals. Therefore, by considering only arterial hypertension, diabetes mellitus (DM), and mixed dyslipidemia as major constituents of the syndrome, we acknowledge that these entities indeed represent significant cardiovascular risk factors. The acquisition and rigorous assessment of these components becomes an imperative necessity, considering the profound implications of their existence, which ultimately can culminate in the emergence of life-threatening pathological conditions, such as acute myocardial infarction and ischemic or hemorrhagic cerebrovascular accidents [10,11,12].

Analyzing each constituent factor of MS is necessary to comprehensively evaluate the patient’s pathological status. Simultaneously, clinical, biological, and functional monitoring of patients becomes mandatory.

Bronchial obstructive pathology and MS may progress independently, each condition evolving autonomously. However, it is widespread for patients to present a primary underlying disease and one or more comorbidities simultaneously. The potential association between bronchial obstructive disorders and MS has acquired profound significance in this context. MS comprises several pathological conditions, conferring this association a unique and noteworthy character [13,14].

Patients with chronic obstructive bronchial conditions encountering specific elements of the MS often experience a more rapidly unfavorable progression. The most severe consequences are undoubtedly attributed to the presence of high blood pressure, DM, obesity, and atherogenic dyslipidemia. Nonetheless, the other pathological elements within the syndrome should not be underestimated [15,16].

Recently, numerous extensive studies have consistently demonstrated a correlation between MS and impaired lung function across different age groups [17]. Individual constituents of MS were demonstrated to have an independent association with this undesirable process. Among these factors, abdominal obesity emerged as the most strongly correlated with the observed outcome [18].

Inflammation linked to increased fatty acids, elevated leptin, reduced adiponectin levels, and alterations of the systemic concentration of growth hormone are some potential pathways involved in airway hyper-reactivity and obstruction [19,20,21,22,23,24,25,26]. Furthermore, exposure to heightened insulin levels during the early gestational period or later in life may instigate morphological or functional alterations in airway smooth muscle [27,28,29].

Compromised lung function is increasingly acknowledged as a potential prognostic indicator for premature mortality [18]. The rate of decline in pulmonary function serves as a valuable insight, offering the opportunity to intervene proactively before reaching irreversible stages and disability. Recent studies concluded that a decrease in FEV1 rates ranging from 30 to 60 mL/year increases the associated risk of death, and this interrelation becomes particularly significant for declines exceeding 90 mL/year [30].

The motivation behind this research was based on the necessity to assess key clinical, biological, and functional parameters found in patients who concurrently manifest obstructive bronchial pathology and MS. Efficiently managing behavioral risk factors is crucial in correcting imbalances and enhancing the quality of life for patients with chronic conditions, including obstructive pulmonary disease.

Especially with advancing age, clinical studies highlight a significant impact on nutritional imbalances generated by the quality of nutrition and unhealthy lifestyle, imbalances that complicate chronic pathologies and increase the risk of premature death [31,32]. As a result, we considered it appropriate to evaluate the clinical data of some generally older patients (over 45 years old) with obstructive pulmonary diseases or MS in correlation with a series of crucial behavioral risk factors specified in the specialized literature aimed to assess the quality of food, quality of rest, physical activity, efficient hydration of the body, and consumption of tobacco or alcoholic beverages. This correlation offers the possibility to identify some unhealthy aspects with significant impact and to be able to issue effective recommendations for efficient balancing.

The present study aims to evaluate respiratory function using spirometry while concurrently examining the associated risk of MS concerning patients’ dietary habits and lifestyle choices. Eating patterns and the presence of unhealthy behaviors, such as smoking, alcohol consumption, sleep quality, and types of physical activity, were systematically observed. Additionally, various biochemical parameters for MS, including fasting blood glucose, high-density lipoprotein cholesterol, and triglycerides, were assessed alongside pulmonary function. Blood pressure readings and anthropometric measurements were also performed.

A secondary objective was to quantify the impact of modifiable behavioral risk factors on metabolic imbalances and respiratory function in the patients included.

The evaluation of behavioral factors (diet, lifestyle) was carried out based on previously used validated questionnaires, and the impact of MS on pulmonary function was determined by evaluating the respiratory function of the patients and reporting to the group of patients without MS.

## 2. Materials and Methods

### 2.1. Study Design and Participants

We conducted this epidemiological non-interventional cross-sectional and case-control study between 1 January and 26 May 2023 in the southwestern region of Romania. We followed the ethical principles outlined in the updated Declaration of Helsinki and obtained the approval of all relevant local Ethics Committees:

1. The Ethics and Deontology Committee within the University of Medicine and Pharmacy from Craiova (number 143/04.07.2022),
2. The Clinical Emergency Country Hospital from Craiova (number 3000/17.01.2023) and
3. The Clinical Hospital of Infectious Diseases and Pneumophtisiology‚ Victor Babeș’ from Craiova (number 7488/22.05.2023).

To evaluate the impact of MS on lung function and respiratory dysfunctions, we used two groups of patients: patients with known chronic lung diseases in which the presence of MS was also evaluated (cohort 1) and patients with known and monitored MS (diabetic patients) in which lung function was evaluated (cohort 2). Patients without MS were established as the control group against which the report was made. In addition, the respiratory function was evaluated according to the number of MS criteria fulfilled in patients with MS (between 3 and 5).

We evaluated 160 consecutive adult patients enrolled from two different clinical departments who expressed their written consent and were assured regarding the confidentiality of their personal data according to the General Data Protection Regulations. Each patient was assigned a unique reference number within the study to ensure data anonymization.

The inclusion criteria were age 18 years and above, residence in Romania, and absence of serious pathologies, such as cancer and advanced mental illnesses that could deprive the patient of discernment and would not have adequately allowed the patient to answer the questionnaires for the assessment of eating behavior and lifestyle.

This study aimed to assess the associated risk of MS in patients with obstructive lung diseases, directly correlating with dietary habits and lifestyle. Investigating these aspects will provide valuable data about this interplay and contribute to obtaining a more effective management plan for this category of patients.

For all the subjects participating in the study, the reason for hospitalization was routine clinical assessment of the chronic condition. Clinical periodic evaluations are critical in managing patients with chronic obstructive pulmonary diseases. These assessments provide the opportunity to monitor the progression of the disease and adjust the therapeutic plan based on changes in the patient’s health status. By conducting this evaluation regularly, exacerbations of the disease can be detected early, symptoms can be evaluated, and treatment strategies can be adapted to improve the patient’s quality of life and prevent complications. Additionally, these evaluations offer the opportunity to provide counseling and medical education to the patient, thereby contributing to proper disease management and improving long-term clinical outcomes. The study cohort consisted of patients admitted to the hospital because this choice allowed us to perform a meticulous paraclinical exploration to evaluate the targeted parameters accurately.

We conducted a bidirectional analysis, so from the beginning, we included patients in two main groups, as follows:

Cohort 1 consisted of 80 consecutive patients previously admitted to the hospital for obstructive lung disease: chronic obstructive pulmonary disease (COPD) or bronchial asthma. The patients were introduced from two Pneumology clinics (Leamna Clinical Pneumophthisiology Hospital and The Clinical Hospital of Infectious Diseases and Pneumophthisiology V‘ictor Babeș’ from Craiova).

The latest criteria for diagnosing obstructive lung diseases were used: for COPD, we followed the Global Initiative for Chronic Lung Diseases (GOLD) 2023 guidelines, and for Bronchial Asthma, we adhered to the Global Strategy for Asthma Management and Prevention 2023 [33].

Cohort 2 consisted of 80 consecutive patients with hospital admissions for type 2 diabetes (based on the American Diabetes Association guidelines) enrolled from The Department of Diabetes and Nutritional Diseases of the Clinical Emergency Country Hospital from Craiova. All of them met the criteria for MS.

Diagnostic criteria for MS were determined by considering the National Cholesterol Education Program Adult Treatment Panel III standards. According to these standards, MS is characterized by the presence of at least three of the following components [34]:

- Elevated waist circumference (≥88 cm for women, ≥102 cm for men).
- Elevated triglycerides (≥150 mg/dL) or drug treatment for high triglycerides levels.
- High-density-lipoprotein (HDL) cholesterol (<40 mg/dL for men, <50 mg/dL for women) or drug treatment for low HDL cholesterol levels.
- Elevated blood pressure (systolic ≥ 130 mmHg or diastolic ≥ 85 mmHg) or treatment for hypertension.
- Elevated fasting glucose (≥100 mg/dL) or treatment for elevated glucose.

All patients included in the study were evaluated at the time of inclusion to determine whether they met the criteria for an MS diagnosis.

The exclusion criteria for both cohorts consisted of:

- Conditions and symptoms that overlapped contraindications for performing spirometry;
- Clinical conditions, physiological or pathological, that can result in abdominal volume expansion and subsequently impact the measurement of abdominal circumference and/or body weight;
- The inability to maintain an upright position without support to be weighted;
- Severely altered clinical condition incompatible with the patient’s ability to comply with the required evaluation steps. This decision was prompted by the need for patients to undergo spirometry (a demanding test), be mobilized for anthropometric measurements, and be able to communicate effectively to complete questionnaires;
- Patients experiencing episodes of respiratory pathology exacerbation or diabetic ketoacidosis. This choice is justified by the observation that exacerbations of lung diseases are generally associated with a decrease in lung volumes measured by spirometry and increased severity levels of respiratory dysfunction in comparison to the baseline values of our patients. Furthermore, periods of diabetes decompensation correlate with substantial changes in biological markers.

These criteria were applied to ensure the safety and accuracy of the study.

### 2.2. Medical History, Anthropometric, and Blood Pressure Measurements

All cases were evaluated with detailed medical history focusing on relevant medication usage, such as lipid-lowering agents and hypoglycemic and antihypertensive drugs. The obtained data underwent verification and were subsequently recorded in the database with coding ‘yes’/‘no’ in the corresponding fields.

The smoking status was noted based on the following risk categories: never smoker, former smoker (currently abstinent), and active smoker.

We continued with physical examination and anthropometric measurements, including height and weight; the last two were conducted with light clothing and no footwear.

The weighing was performed using a medically certified scale that conforms to electromagnetic compatibility standards: OMRON Body Composition Monitor BF511 HBF-511T-E/HBF-511B-E (OMRON HEALTHCARE Company; Kyoto, Japan). Height was measured with a stadiometer with patients in the upright position. Body mass index (BMI) was determined for each individual according to the formula BMI = weight (in kilograms)/height squared (in meters) [35,36]. The patients were subsequently classified based on their BMI values according to the World Health Organization’s specifications [37]. The abdominal circumference assessment was made during full expiration with a flexible, non-stretchable tape while patients were standing upright with their feet close and their arms on the sides. Blood pressure was also checked using a validated automated electronic device (OMRON X3 Comfort HEM-7155-EO; Omron Company; Kyoto, Japan). This evaluation was made in a silent hospital room with the patient seated calmly and quietly for at least 10 min in a chair with back support and with feet on the floor, uncrossed, without having drunk caffeine or alcoholic beverages, smoked cigarettes, or performed any physical exercise for at least 30 min prior. The determinations were performed following the recommendations outlined by the American Heart Association (AHA).

### 2.3. Blood Samples—Biochemical Parameters

Ten milliliters of venous blood were collected from each patient in a vacutainer using classic protocol to determine fasting blood glucose, triglycerides, and HDL cholesterol. The processing method used an automatic analyzer—BioSystems A15 (BioSystems Company, Barcelona, Spain). The resulting values were then assigned to each patient’s corresponding order number and recorded in the database. It is essential to mention that including hospitalized patients allowed us to ensure that blood samples were collected under optimal conditions regarding the preceding fasting period.

### 2.4. Pulmonary Function Test—Spirometry

All enrolled patients underwent spirometry using the same Spirolab IV (Medical International Research Company; Roma, Italy) machine. The device obtained a conformity certificate according to European safety standards EN 60601-1 and ensured electromagnetic compatibility within the limits of EN 60601-1-2; it is available online at https://spirometry.com/media/documents/MIR-Linea-Standard-Spirolab-en.pdf, accessed on 10 October 2023. To minimize any risk of contamination, single-use turbines and mouthpieces were utilized for each individual. During all the evaluation procedures, a single spirometer was used following the optimal operating conditions stipulated by the manufacturer. The same operator guided all cases, and a resident physician was trained in pulmonology. For the correct execution of the test and the interpretation of the results, we conformed to the American Thoracic Society (ATS)/European Respiratory Society (ERS) guidelines for spirometry standardization. The assessment of respiratory function adhered to criteria outlined by international standards. For example, we made sure that patients had not used short-acting inhaling drugs within the preceding 4 h before spirometry.

Furthermore, the cessation of long-acting β-agonist bronchodilators or oral therapy with aminophylline or slow-release β-agonists was enforced 12 h before the test. Additionally, we ensured that patients did not smoke in the last hours before the lung testing. The absolute values and the percentage of predicted values for forced expiratory volume in one second (FEV1) and forced vital capacity (FVC) were recorded. Additionally, the FEV1/FVC ratio was calculated for every single patient. Subsequently, we quantified the spirometric patterns encountered, including normal pattern, obstructive ventilatory dysfunction (DVO), restrictive ventilatory dysfunction (DVR), and mixed ventilatory dysfunction (DVM), along with their corresponding degrees of severity [38].

### 2.5. Assessment of Lifestyle and Diet

All patients were requested to respond to a standardized questionnaire to systematically observe different aspects of lifestyle and dietary habits, emphasizing eating routines and the presence of unhealthy behaviors, including queries about smoking habits, alcohol consumption, type of physical activity, and respiratory noxious exposure. Noxious exposure refers to contact with harmful or toxic substances that can negatively impact health, mainly through inhalation. To facilitate accurate identification of affirmative exposure, the person guiding the questionnaires provided detailed examples of professional exposure to respiratory hazards, including exposure to chemical fumes, particulate dust, various gases, biological agents, and smoke. A validated questionnaire was used to evaluate the behavioral risk factors (the questionnaire was validated for studies that assess the negative impact caused by excessive consumption of junk food and sweetened beverages) [39,40]. The questionnaire quantified the consumption and frequency of quality nutritious foods that also have protective factors (fibers, probiotics, antioxidants, vitamins, minerals, essential amino acids, essential fatty acids), ultra-processed foods and drinks with a high content of saturated fats and refined carbohydrates (junk food, sweetened drinks), the consumption of tobacco and alcoholic beverages, physical activity, and sleep duration, which affect the patient’s quality of life.

The survey was conducted using an online platform, Google Forms, for registration. A physician underwent rigorous training to ensure the accuracy of recording responses and the identification number assigned to each patient in the study. That person was responsible for verbally presenting each survey question to the participants, clearly enumerating response options, and ultimately marking the checkbox corresponding to each received answer, guaranteeing the data’s reliability.

### 2.6. Statistical Analysis

The background characteristics of the subjects included in the study were described using descriptive statistics. Categorical variables were presented using absolute frequencies (n) and relative frequencies (%). Principal component analysis (PCA) was employed to identify possible associations between behavioral risk factors (diet, physical activity, tobacco use), gender, and anthropometric measurements (BMI).

Variable parameter correlations were established via principal component analysis using XLSTAT 2023.1.4. by Lumivero (Denver, CO, USA) through Pearson correlation [41,42].

Receiver operating characteristics (ROC) curve analysis, quantified by the area under the ROC curve (AUC), was utilized to assess the values of the indicators (age, fasting blood glucose, BMI, and TGL) for predicting MetS. Youden’s index was employed to determine the optimal cut-off point of each index.

A probability value of *p* <0.05 signifies statistically significant differences, while r represents the correlation index.

## 3. Results

### 3.1. Assessment of Behavioral Risk Factors (Diet and Lifestyle)

#### 3.1.1. Cohort 1

In the case of patients from cohort 1, 36.25% were female (n = 29) and 63.75% male (n = 51), as can be observed in Table 1. Most patients were between 56–75 years of age (65%). Of the entire cohort, 53 patients, constituting 66.25% of the sample, had MS according to the diagnostic criteria applied. Among these, 60.37% were male (32 individuals). The distribution of patients by residential areas was approximately equal between rural and urban; 51.25% live in the city. Most patients had a low educational level; 53.75% had primary education without a baccalaureate diploma, and only 5% had higher education. Regarding occupational status, over 66% of patients were retired, and approximately 15% were employed. Over 36% of patients were former smokers, and over 32% were active smokers. Most smokers were males (Table 1). More than 43% of the patients declared that they worked in a toxic environment, most of them being men. Regarding alcohol consumption, the majority of patients stated that they do not consume alcohol (more than 63%), and only 12.5% declared that they are chronic drinkers (exclusively males). According to the centralized data in Table 1, the most reported health risk factors were smoking and exposure to pollutants in the case of patients from cohort 1.

The characteristics of cohort 1 of patients according to the MS score and the results of the spirometric evaluation of pulmonary function are shown in Table 2.

**Figure 1:**
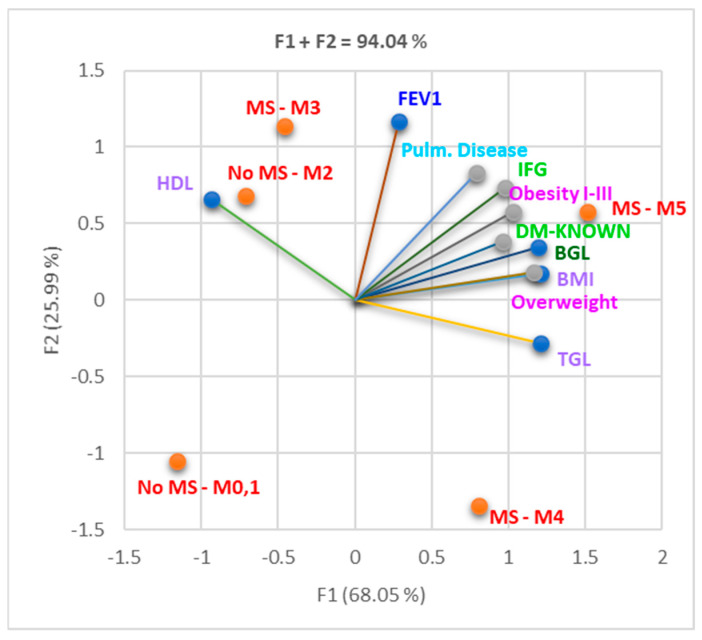
Correlation bi-plot between MS parameters and FEV1, pulmonary disease (asthma/COPD), and diabetes. Legend: No MS = the diagnostic criteria for MS were not met; MS 0–5 = number of MS criteria present: 0–5; BGL = blood glucose level; IFG = impaired fasting glucose; Pulm. Disease = pulmonary disease (asthma/COPD); FEV1 = forced expiratory volume in one second; DM known = patient with a past diagnosis of diabetes mellitus.

A total variance of 94.04% was recorded between the analyzed parameters displayed on the first two axes, where the greatest part belonged to F1, including No MS and MS-5; HDL and FEV1, MS-3, and MS-4 correlated more with F2 (Figure 1).

The Correlation Matrix (Supplementary Material, Table S1) shows a substantial correlation between BGL and BMI, obesity, overweight, and IFG (r = 0.981, r = 0.933, r = 0.954, r = 0.908, *p* > 0.05). Confirmed DM diagnosis (DM known) was substantially correlated with obesity and overweight (r = 0.958, r = 0.936, *p* < 0.05). Pulmonary disease (asthma and COPD) significantly correlated with obesity (r = 0.946, *p* < 0.05). BMI values were remarkably correlated with TGL and BGL (r = 0.921, r = 0.981, *p* < 0.05).

**Figure 2:**
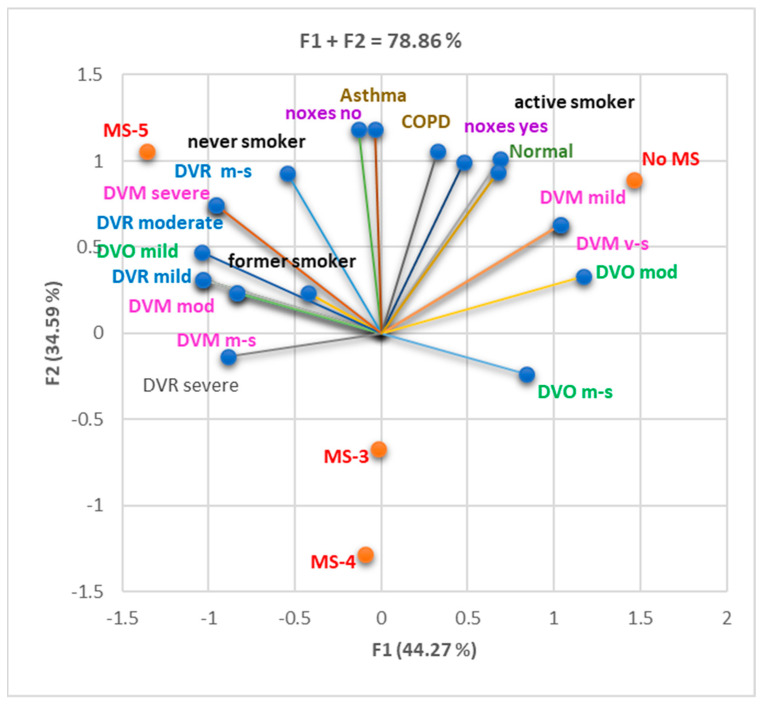
Correlation bi-plot smoker status, respiratory exposure to noxious substances, pulmonary disease (asthma/COPD), and respiratory dysfunctions with different grades, evaluated by spirometry. DVO = obstructive respiratory dysfunction; DVO m-s = obstructive respiratory dysfunction moderate–severe; DVR = restrictive respiratory dysfunction; DVR m-s = restrictive respiratory dysfunction moderate–severe; DVM = mixed respiratory dysfunction; DVM m-s = mixed respiratory dysfunction moderate–severe; DVM v-s = mixed respiratory dysfunction very severe; No MS = not meeting the diagnostic criteria for MS; MS-3–5 = diagnosis of MS and the number of criteria met; Noxes no = affirmative no history of exposure to respiratory noxious agents; Noxes yes = affirmative history of exposure to respiratory noxious agents.

The PCA bi-plot from Figure 2 displays the correlations between smoking status and respiratory exposure to noxious substances, pulmonary disease (asthma/COPD), and respiratory dysfunctions with different grades evaluated by spirometry. The variable parameters displayed between the F1 and F2 axes lead to a total variance of 78.86% (Figure 2).

Figure 2 shows that COPD was substantially correlated with noxious exposure (r = 0.991, *p* < 0.05), while asthma correlated with the absence of exposure (r = 0.997, *p* < 0.05). The active smoker status showed a good correlation (0.900 > r > 0.800, *p* > 0.05) with COPD and DVM mild and very severe (Figure 2), while former smokers and never smokers with severe DVR and asthma. Whether mild or severe, DVR also correlates well with noxious exposure. Moreover, asthma moderately correlated with active smoking and noxious exposure (r < 0.800, *p* > 0.05). Moderate correlations were evidenced between DVM, DVO, and DVR and both tobacco and noxious exposure (*p* > 0.05). The presence of COPD and mixed ventilatory dysfunction, in particular, was strongly influenced by exposure to pollutants and the habit of smoking.

**Figure 3:**
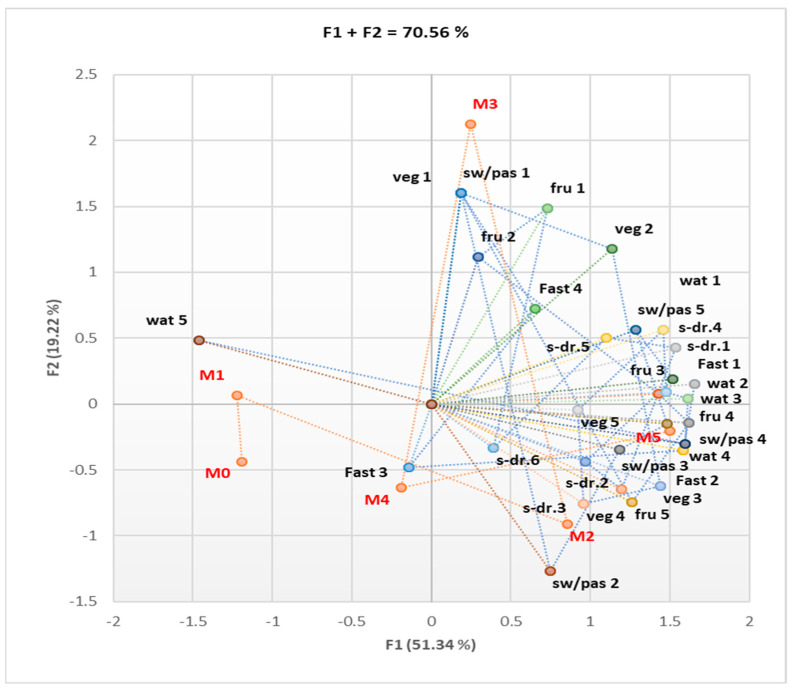
Symmetric bi-plot for the association of various diet habits in different numbers of MS criteria present (M0–5); fast = fast food; sw/pas = sweets/pastries; s-dr = sweet drinks; veg = vegetable products; fru = fruits; wat = water.

We quantified the number of criteria met for the MS and the frequency of consumption of some food products, as follows: 1—very rare or no consumption; 2—consumption 2–3 times a month; 3—consumption once a week; 4—consumption 2–3 times a week; 5—daily consumption of fast food, sweets, and pastries. In the case of sweetened and carbonated drinks, the following quantification of consumption frequency was used: 1—very rare or no consumption; 2—consumption 2–3 times a month; 3—consumption once a week; 4—consumption 2–3 times a week; 5—daily consumption of one portion (330 mL/portion); 6—daily consumption of more than one portion. The quantification of the consumption of vegetables and fruits was conducted as follows: 1—consumption of more than three portions of 100 g per day; 2—consumption of three portions per day; 3—consumption of two portions per day; 4—consumption of one portion per day; 5—consumption very rarely or no consumption. Quantification of water consumption was as follows: 1—consumption over 3 L per day; 2—consumption of 3 L daily; 3—consumption of 2 L daily; 4—consumption of 1 L daily; 5—consumption below 1 L daily. As seen from Figure 3, patients with many MS criteria fulfilled (M5) tend to consume sweet products, pastries, and sweetened drinks more frequently and have a lower tendency to consume vegetables and fruits and even correctly hydrate.

**Figure 4:**
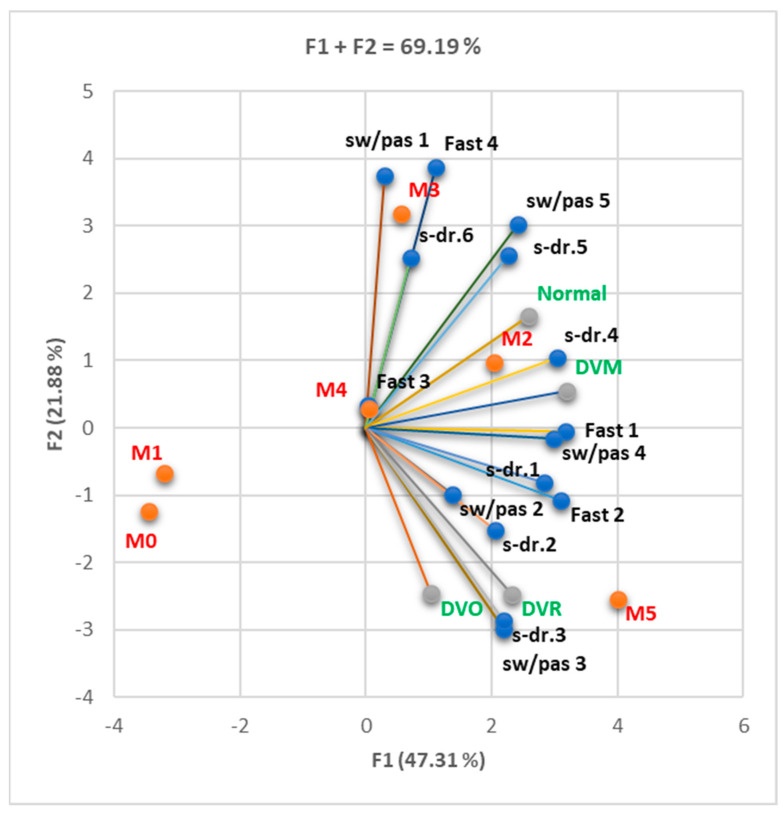
The correlations between spirometry pattern (DVM, DVO DVR, and normal), unhealthy diet (fast food, sweets, pastries, and sweet drinks consumption), and the number of MS criteria present (M0–5). M3, sw/pas 1, and Fast 4 are linked with F2. Most variables are related to F1. Legend: fast = fast food; sw/pas = sweets/pastries; s-dr = sweet drinks.

The Correlation Matrix (Supplementary Material, Table S2) shows considerable statistical correlations between DVM and increased consumption of sweets, pastries, and sweetened drinks (sw/pas 4, s-dr 4; r = 0.916–0.973, *p* < 0.05). DVR strongly correlated with relatively high consumption of sweets and pastries (sw/pas 3; r = 0.821, *p* < 0.05), while normal respiratory function correlated with reduced consumption of fast food products (Fast 1; r = 0.822–0.897, *p* < 0.05). The large number of MS criteria found in an individual was associated with an increase in the frequency of consumption of sweet products, sweetened drinks, and fast food products (Figure 4).

**Figure 5:**
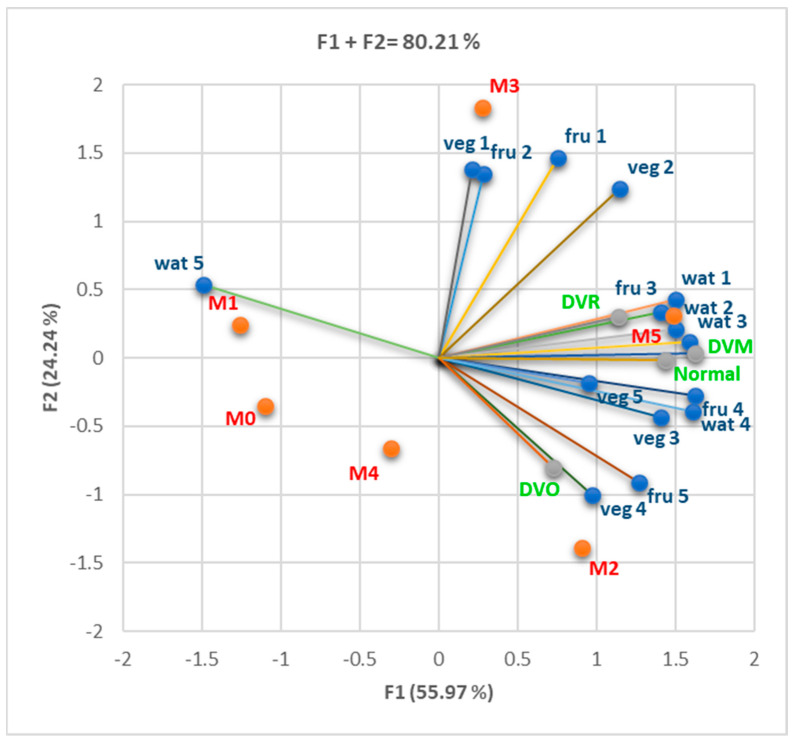
Correlation between spirometry pattern (DVM, DVO DVR, and normal), healthy diet (vegetable products, fruits, water consumption), and the number of MS (MS) criteria present (M0–5). Fru 1–2 and veg 1, 2, and 4 are linked with F2. Most variables are associated with F1. Legend: veg = vegetable products; fru = fruits; wat = water.

We found substantial correlations between DVM, DVR, and relatively low consumption of fruits, vegetables, and water (fru 3, fru 4, veg 3, wat 3, wat 4; r = 0.814–975, *p* < 0.05), as can be seen in Figure 5 and the Correlation Matrix (Supplementary Material, Table S3). Normal respiratory function strongly correlated with increased water consumption (wat 1; r = 0.937, *p* < 0.05). Patients with many fulfilled MS criteria (M5) showed a low tendency to consume vegetables and fruits and a tendency to lower daily water consumption.

**Figure 6:**
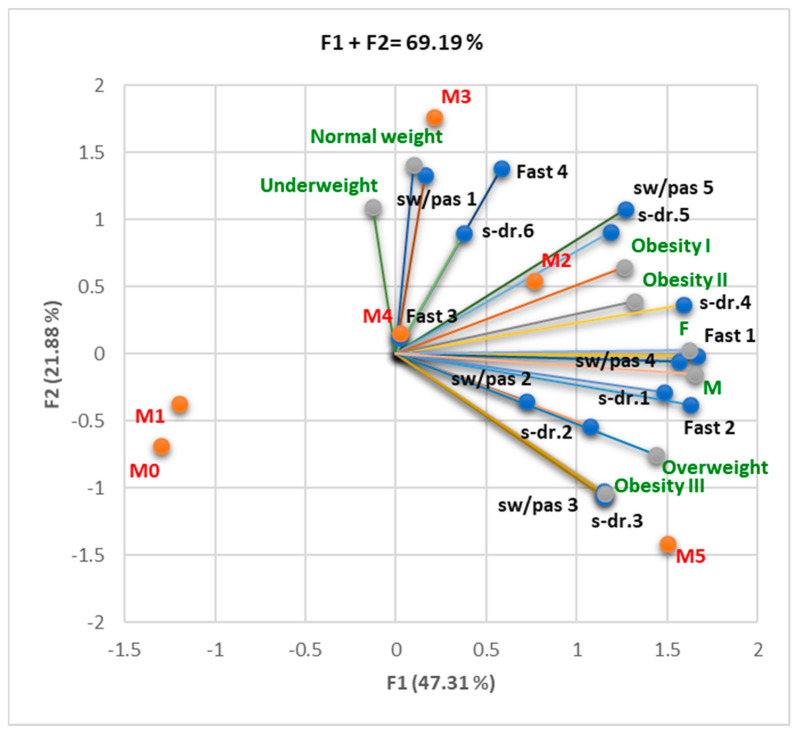
Correlations between BMI classes (underweight, normal weight, overweight, obesity I–III), gender (F and M), unhealthy diet (fast food, sweets, pastries, and sweet drinks consumption), and the number of MS (MS) criteria present (M0–5). F1 is associated with almost all parameters. F2 is related to fast 4 and sw/pas 1, normal weight, and underweight. Legend: fast = fast food; sw/pas = sweets/pastries; s-dr = sweet drinks.

The Correlation Matrix (Supplementary Material, Table S4) shows that overweight and obesity I–III were significantly correlated with increased consumption of sweet products, pastries, and sweetened drinks [sw/pas 5 (*p* < 0.05, r = 0.813), sw/pas 4 (*p* < 0.05, r = 0.896), s/dr.4 (r = 0.812 *p* < 0.05)]. Grad II obesity was substantially correlated with females (r = 0.909) and overweight with males (r = 0. 879, *p* < 0.05). Regarding the diet, both sexes showed significant preferences for relatively high consumptions of sweet or pastry products, as well as sweetened drinks, sw/pas 4 (r = 0.816–0.908, *p* < 0.05), and s.dr.4 (r = 0.896–0.900, *p* < 0.05). There was a strong correlation (Figure 6) between the increased number of MS criteria and the recurrence of unhealthy food consumption (sweets, pastries, and sweetened drinks).

**Figure 7:**
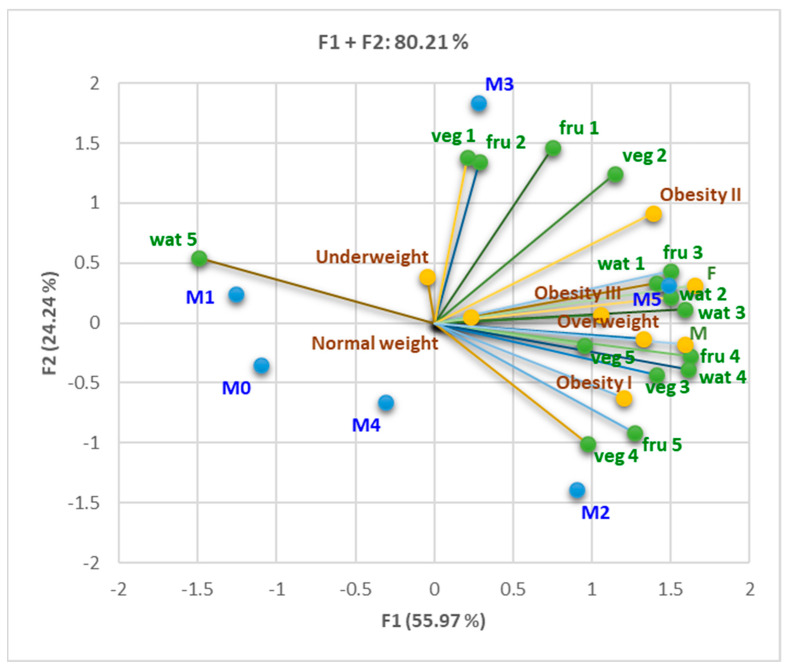
Correlations between BMI classes (underweight, normal weight, overweight, obesity I–III), gender (F and M), healthy diet (fruits, vegetables, and water consumption), and the number of MS (MS) criteria present (M0–5). Water, veg 3, fru 3–5, and M0, 1, and 5 are associated with F1. Gender, M2–4, obesity I and II, and overweight are associated with F2. Legend: veg = vegetable products; fru = fruits; wat = water.

Figure 7 shows that both sexes displayed a significant tendency towards a relatively low daily consumption of vegetables and fruits (1–2 portions per day) and moderate consumption of water (1–2 L per day), with women having a slightly higher consumption tendency compared to men: fru 3 and 4 (r = 0.845–0.926, *p* < 0.05), veg 3 (r = 0.781–0.913, *p* < 0.05), wat 3–4 (r = 0.884–0.975, *p* < 0.05). Being overweight was strongly correlated with moderate consumption of vegetables and fruits (two portions per day) and consumption of 2 L of water daily: fru 3, veg 3, wat 3 (r > 0.900, *p* < 0.05). Grade I of obesity had a strong correlation with the reduced consumption of vegetables and fruits (one portion per day) and with the consumption of 1 L of water daily: fru 4, veg 4, and wat 4 (r = 0.742–0.858, *p* < 0.05). The increase in MS criteria was associated with the tendency to consume fewer vegetables and fruits and less water.

To evaluate the impact of physical activity, the following quantification was used: spr 1 (physical activity/movement for at least one hour daily); spr 2 (physical activity/movement less than an hour daily); spr 3 (physical activity/movement 2–3 times a week); spr 4 (physical activity/movement very rarely); spr 4 (physical activity/movement no).

**Figure 8:**
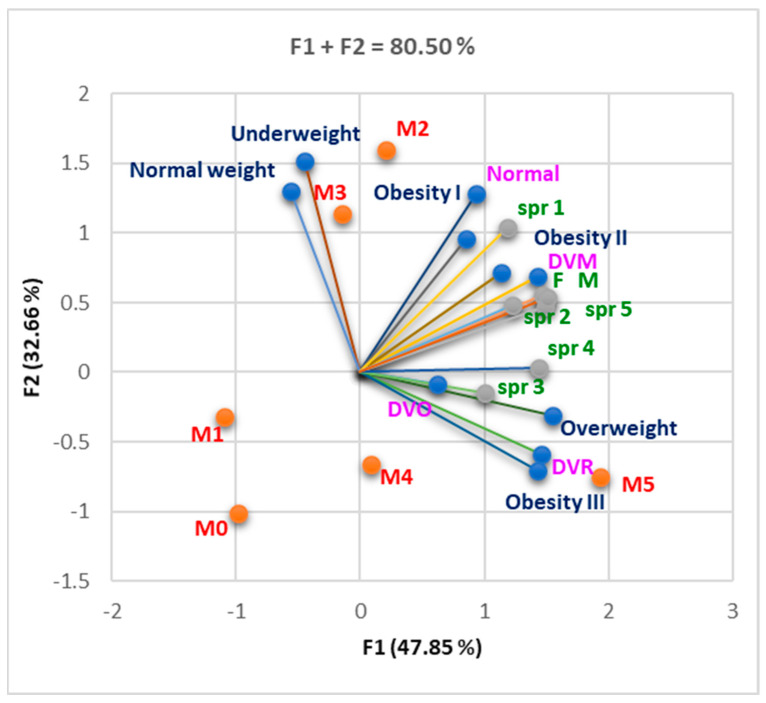
The correlation between BMI status, respiratory dysfunctions, gender, and sport/physical exercises and the number of MS (MS) criteria present (M0–5). F1 is associated with gender, sport practice (1–5), obesity II–III and overweight, DVM and DVO, M0, M1, and M5. F2 is associated with normal weight, normal respiratory status, and underweight. Legend: spr = sport (physical activity).

The Correlation Matrix (Supplementary Material, Table S5) shows that intense physical activity was strongly correlated with normal respiratory function (spr 1, r = 0.917, *p* < 0.05), and reduced physical activity and sedentarism were strongly correlated with overweight (spr 4, r = 0.842; spr 5, r = 0.857, *p* < 0.05). We also found a strong correlation between DVM and total lack of physical activity (spr 5, r = 0.992, *p* < 0.05). A strong correlation existed between DVR and overweight and obesity III (r = 0.937–0.987, *p* < 0.05). Figure 8 shows a tendency towards sedentarism, linked with an increase in the number of criteria fulfilled and a weight increase.

**Figure 9:**
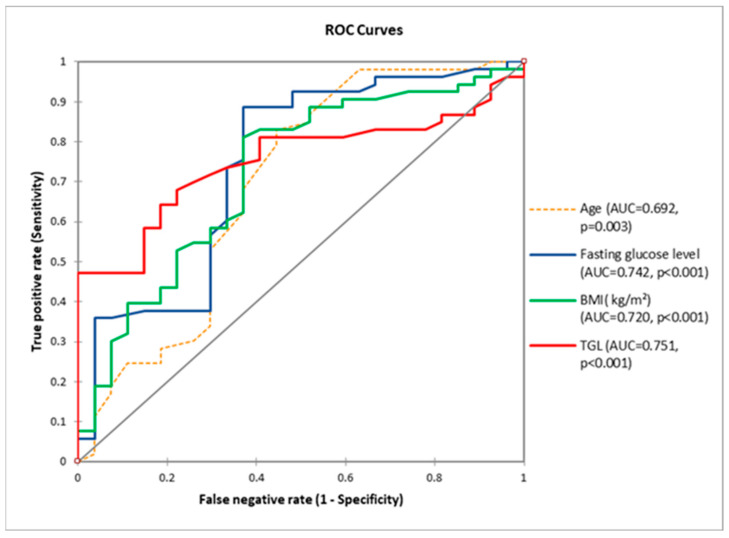
Receiver operating characteristic curves (ROC) for the parameters involved in the risk of MS (AUC = area under the curve).

The optimal cut-off value for age to detect the MS with maximum sensitivity and specificity was 58 years old, with a positive predictive value (PPV) of 78.57% and a negative predictive value (NPV) of 62.50%. The optimal cut-off value for fasting blood glucose was 101 (mg/dL), with a positive predictive value (PPV) of 82.46% and a negative predictive value (NPV) of 73.91%. The optimal cut-off value for BMI was 24.7 kg/m^2^, with a positive predictive value (PPV) of 81.13% and a negative predictive value (NPV) of 62.96%. For TGL, the cut-off value was 109 mg/dL, the positive predictive value (PPV) was 92.59%, and the negative predictive value (NPV) was 49.09% (Figure 9).

#### 3.1.2. Cohort 2

According to the data in Table 3, among the patients in cohort 2, the majority were women (44 persons) compared to 36 men, most of them aged between 46 and 65 years, overweight (38.75%), and with grade I obesity (28.75%). Half of the patients (50%) declared that they had never smoked, 22.5% were active smokers, and 27.5% were former smokers; most of the patients who currently smoke or have smoked were men. More than half of the total number of patients (53.75%) declared that they were not exposed to noxious agents, 12.5% of the patients declared themselves to be chronic alcohol consumers, all of them being male, and 30% stated that they are occasional drinkers. Regarding the educational level, most patients were primary school graduates without a baccalaureate (48.75%); only 17.5% were higher education graduates, 62.5% were retired, 17.5% were employed, and 57.5% were living in an urban environment.

The characteristics of cohort 2 patients according to the MS score and the results of the spirometric evaluation of pulmonary function are shown in Table 4.

**Figure 10:**
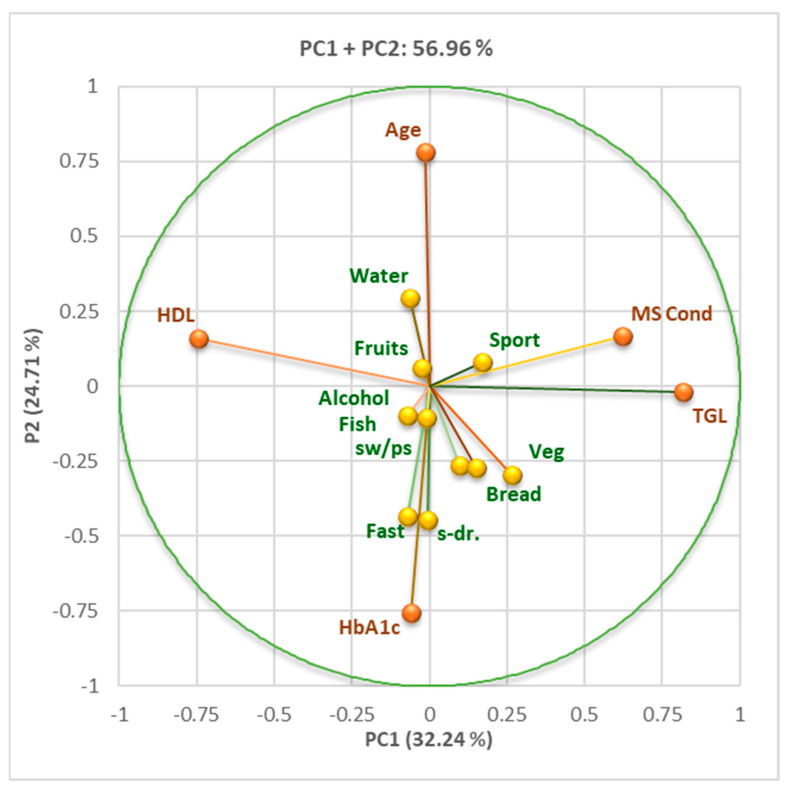
Principal component analysis for age, HDL, HbA1c, TGL, MS Cond, and diet (water, fruits, fish, alcohol, vegetables, bread). Age, HbA1c, and almost all diet aspects are linked with PC2, while TGL and HDL are associated with PC1. Legend: fast = fast food; sw/pas = sweets/pastries; s-dr = sweet drinks; veg = vegetable products; MS Cond = MS conditions met (MS-3–5); HbA1c = glycosylated hemoglobin.

Figure 10 and the Correlation Matrix (Supplementary Material, Table S6) show significant correlations (*p* < 0.05) between HbA1c, fast food, sweet drinks, and bread consumption. Increasing the consumption of fast food products, such as bread and sweetened drinks, can increase glycosylated hemoglobin. We also found a direct correlation between age, HDL cholesterol, and the consumption of vegetable products.

**Figure 11:**
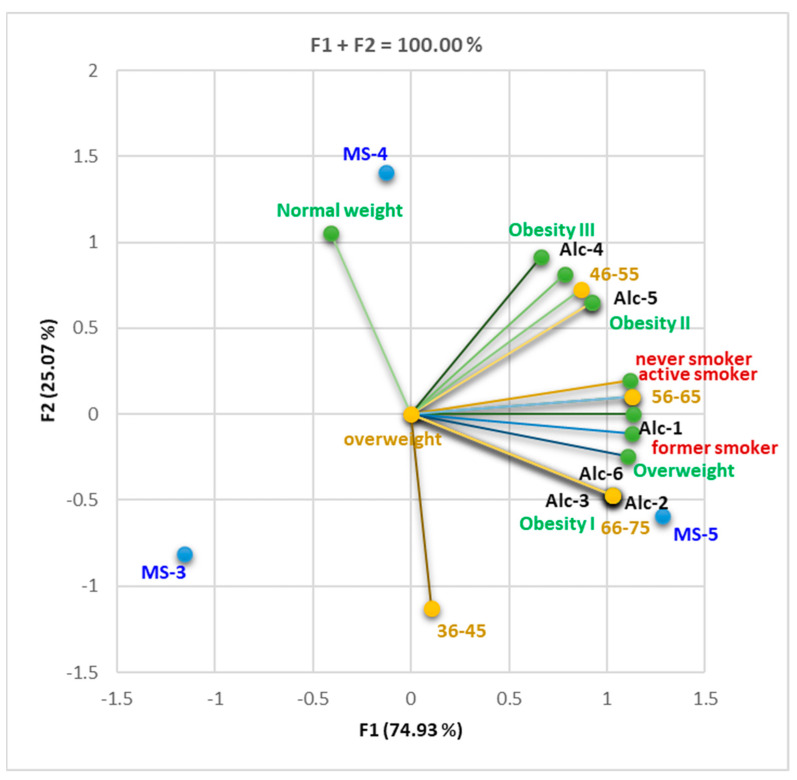
Correlation bi-plot between alcohol and tobacco consumption and BMI status and age in various MS diabetes patients. Legend: MS conditions met (MS-3–5); alc = alcohol consumption (1–6). For alcoholic beverages, the following quantification of consumption frequency was used: 1—very rare or no consumption; 2—consumption 2–3 times a month; 3—consumption once a week; 4—consumption 2–3 times a week; 5—daily consumption of one portion (a portion: 1 glass of wine = 125 mL, 1 glass of soft drink spirit = 50 mL); 6—daily consumption of more than one portion.

F2 was associated with normal weight, obesity III, Alc-4, age of 36–45, and MS-4. All other variables were associated with F1. F1 and F2 ensured 100% of data variation (Figure 11). Age group 66–75 was significantly correlated with Alc-2, 3, and 6 and obesity I, and the 56–65 age group showed a significant correlation with the active smoker status (r = 0.999, *p* < 0.05). Alc-2, 3, and 6 were significantly correlated with obesity I (r = 1, *p* < 0.05), while Alc-5 correlated with obesity II (r = 0.999, *p* < 0.05). Increased alcohol consumption was associated with obesity; the greater the amount consumed, the higher grade of obesity. According to the Correlation Matrix (Supplementary Material, Table S7), the trend of consumption of alcoholic beverages was more pronounced in the age range 56–75 years (r = 0.866–1, *p* < 0.05). There was a strong correlation between the 56–65 age group and active smoking (r = 0.999, *p* < 0.05).

**Figure 12:**
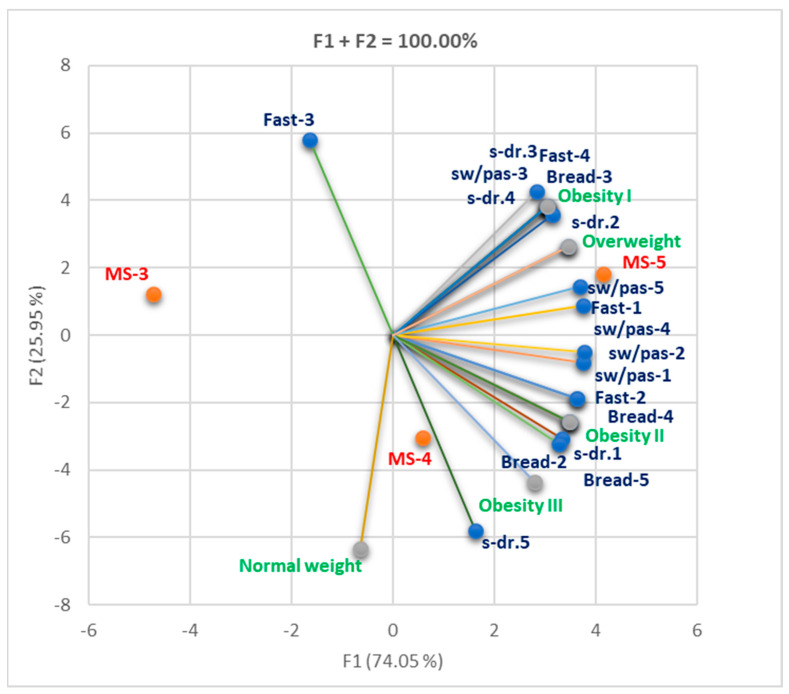
Correlation bi-plot between diet and BMI status in all MS-3–5. F2 is associated Fast 3, s-dr.5, normal weight and MS-4, while F1 is associated with MS-5, overweight, obesity I–III, and almost all types of diets. Legend: fast = fast food; sw/pas = sweets/pastries; s-dr = sweet drinks; MS conditions met (MS-3–5). Bread consumption was quantified as follows: 1—very rarely or not at all; 2—1–4 slices daily; 3—5–7 slices daily; 4—8–12 slices daily; 5—over 12 slices daily.

We found that Fast 4, s-dr. 3,4 and bread 4 significantly correlate with obesity I; the same observation is available for s-dr. 2 and overweight and also for bread 4,5 and obesity II (r = 0.999, *p* < 0.05, Table S8). Increasing consumption of fast food products, sweet drinks, and bread led to an increase in excess weight (Figure 12).

### 3.2. Evaluation of the Impact of MS on Pulmonary Function

#### 3.2.1. Cohort 1

We analyzed the influence of the presence of MS on respiratory function in all 160 subjects. Patients without MS were established as a control group. We followed the degree of respiratory dysfunctions, their severity, the absolute values, and the percentage of predicted values for forced expiratory volume in one second (FEV1) recorded in patients with MS compared to patients without it. The control group represents patients from cohort 1 without MS. Compared to the control group, the report was made for the patients with MS from cohort 1. In the case of cohort 2, the analysis was made on the two combined cohorts, where the patients without metabolic syndrome from cohort 1 represent the control group. Moreover, for cohort 2, we correlated the presence of pulmonary dysfunctions with the number of MS criteria met, considering that all patients in this case were diagnosed with MS.

**Figure 13:**
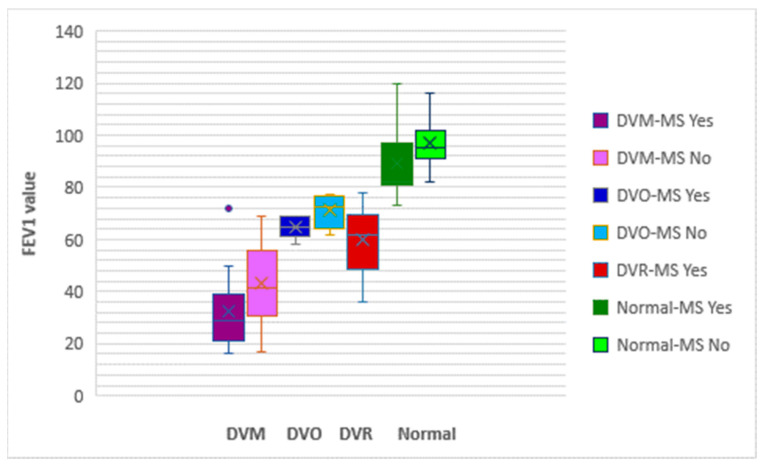
Box and whisker graph of the risk of MS associated with the degree of severity of obstructive pulmonary disease for cohort 1. MS = metabolic syndrome; DVM = mixed respiratory dysfunction; DVO = obstructive respiratory dysfunction; DVR = restrictive respiratory dysfunction; FEV1 = forced expiratory volume in one second.

Figure 13 shows a greater impairment of lung function in patients with MS. The average values of FEV1 are lower in patients with MS than those without MS. We also found a higher severity of pulmonary dysfunction in the presence of MS.

**Figure 14:**
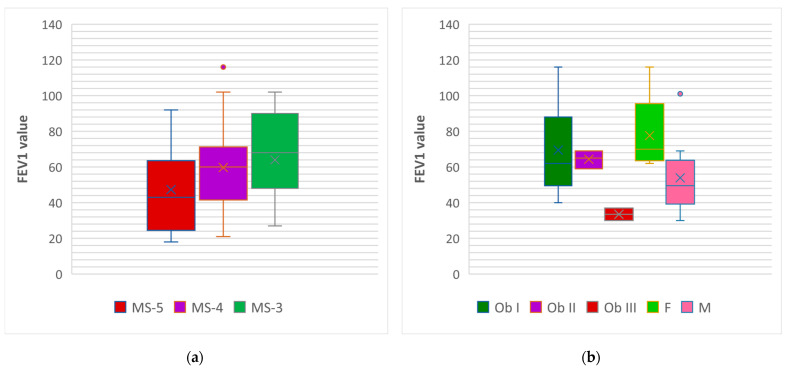
Box and whisker graph of the risk of pulmonary function impairment associated with: (**a**) the number of criteria met for the MS (MS-3, MS-4, MS-5); (**b**) the degree of obesity (Ob I, II, III) and gender (F,M) for cohort 1; FEV1 = forced expiratory volume in one second.

We found that the average value of FEV 1 worsened in correlation with the number of criteria for MS (Figure 14a); the lowest values were in patients who met the maximum criteria (MS-5). A reduction in the average FEV 1 was observed with the increasing degree of obesity; the patients with obesity degree III showed the lowest values (Figure 14b). Moreover, in cohort 1, male patients showed much lower values of the average FEV 1.

#### 3.2.2. Cohort 2

**Figure 15:**
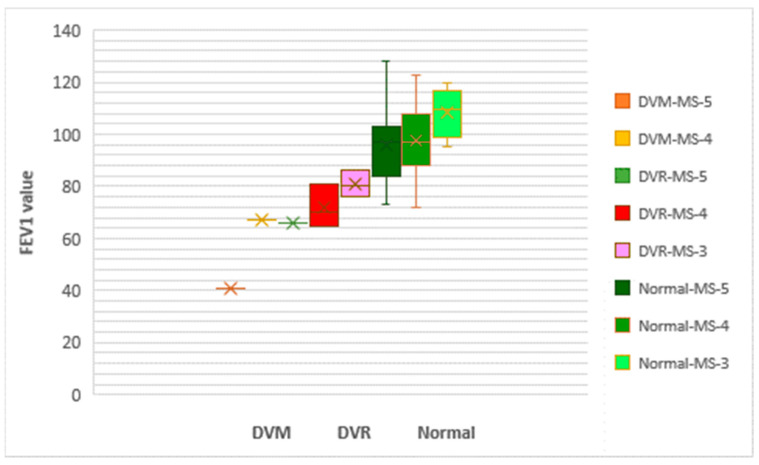
Box and whisker graph of the risk of MS associated with the degree of severity of obstructive pulmonary disease for cohort 2. MS = metabolic syndrome; DVM = mixed respiratory dysfunction; DVO = obstructive respiratory dysfunction; DVR = restrictive respiratory dysfunction; FEV1 = forced expiratory volume in one second.

In the case of MS patients from cohort 2, our analysis of pulmonary function was based on the number of MS criteria that were met. We found that pulmonary function impairment increased with the number of fulfilled MS criteria (Figure 15).

#### 3.2.3. Both Cohorts Analyzed Together

**Figure 16:**
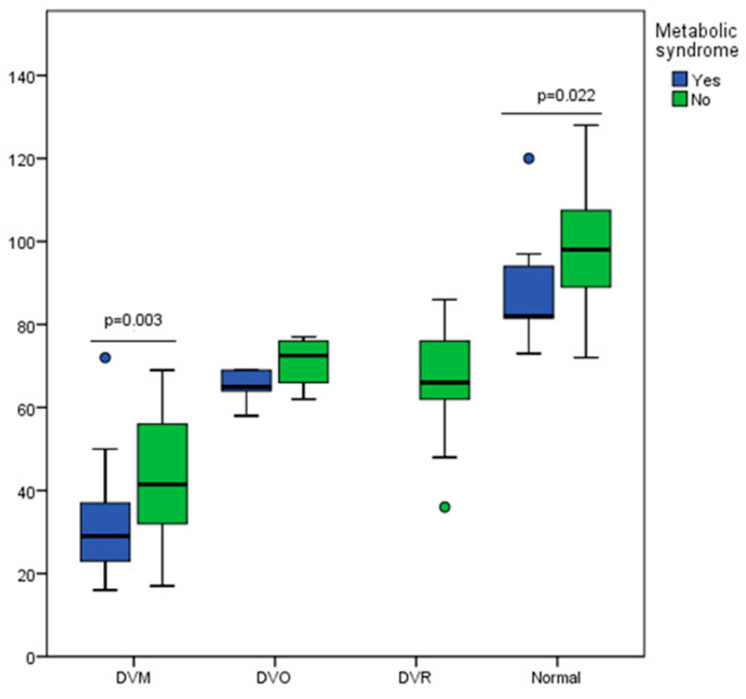
Box and whisker graph of the risk of MS associated with the degree of obstructive pulmonary disease severity for united cohorts. MS = metabolic syndrome; DVM = mixed respiratory dysfunction; DVO = obstructive respiratory dysfunction; DVR = restrictive respiratory dysfunction.

An evaluation of lung function and respiratory dysfunctions was also conducted within the groups of patients (cohort 1 and cohort 2, Figure 16).

Figure 16 shows that, in the combined groups, patients with MS had much lower mean FEV1 values, and the severity of respiratory dysfunction was much more pronounced in patients with MS compared to patients without it.

**Figure 17:**
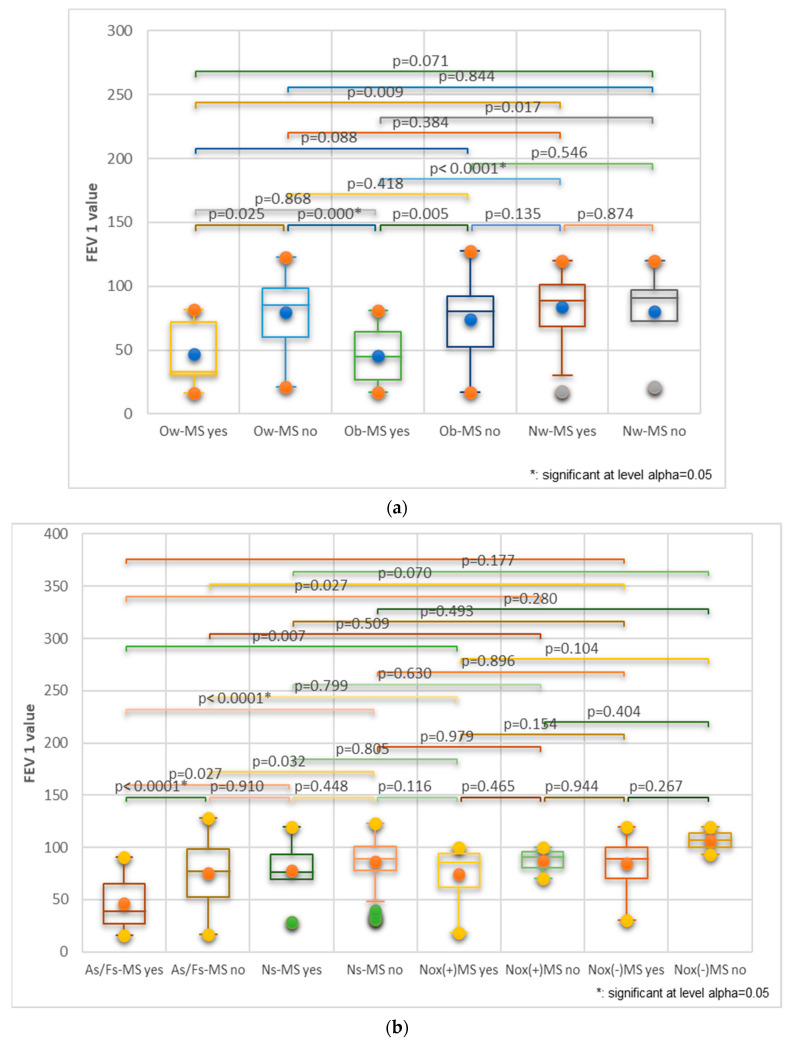
Kruskal–Wallis graph illustrating the increased risk for lung function impairment in united cohorts when MS is associated with (**a**) overweight and obesity and (**b**) smoking and exposure to respiratory noxious. Legend: MS = metabolic syndrome; Ow = overweight; Ob = obesity; Nw = normal weight; As/Fs = active/former smokers; Ns = never smokers; Nox = respiratory noxious; (+) = yes; (-) = no; FEV1 = forced expiratory volume in one second; Bonferroni corrected significance level = 0.0033.

Moreover, the main risk factors for chronic obstructive pulmonary diseases have a more significant influence in association with MS than without it (Figure 17).

Thus, Figure 17a shows that obese patients with MS had the highest risk for lung function impairment, following, in decreasing order, the overweight and normal-weight MS patients. Significant differences could be noted between obese and normal-weight MS patients (*p* < 0.0001). Figure 17b illustrates that MS associated with smoking and respiratory noxious exposure considerably increased the risk for impaired respiratory function. It can be noted that, in smokers, MS substantially increases the risk for obstructive respiratory disease compared to those without MS (*p* < 0.0001).

## 4. Discussion

### 4.1. Involvement of Weight, Diet, and Metabolic Factors in the Two Cohorts

Weight gain, particularly when accompanied by dyslipidemia, might worsen the evolution of specific respiratory pathologies such as asthma or COPD. Excessive weight can impose pressure on lung function, impairing respiratory mechanics. It can manifest as increased dyspnea and reduced lung capacity, exacerbating respiratory conditions. Additionally, obesity is often linked to systemic inflammation, further complicating respiratory function. Hence, addressing weight management and metabolic factors is crucial in controlling and preventing respiratory disorders.

Furthermore, diabetes mellitus might adversely affect respiratory function through various possible mechanisms, including microvascular complications, autonomic neuropathy, and altered immune response. These factors can impair gas exchange and tissue oxygenation, contributing to the progression of respiratory pathology (Figure 1, Table 2).

Figure 1 illustrates that the decline in forced expiratory volume in one second (FEV1) in patients with obstructive lung diseases (asthma/COPD) and diabetes is accentuated among subjects meeting the maximum number of MS criteria and among overweight and obese individuals.

Within the group of patients with lung diseases, we noted a more pronounced tendency of respiratory impairment in patients with severe excess weight and MS. We can hypothesize, based on the statistical analysis, that a sedentary lifestyle and poor dietary quality could have a detrimental impact on the severity of respiratory dysfunctions.

We also found that the aggravation of the MS and respiratory dysfunctions is strongly correlated with an increased tendency to consume some unhealthy foods (such as sweets, pastries, and sweetened drinks) and also with a reduced tendency to consume some nutritious foods (vegetables and fruits), along with inadequate hydration. Additionally, a negative impact on lung function was also noted concerning smoking, exposure to respiratory noxious agents, and a sedentary lifestyle.

Comparing the two analyzed groups, we noted that the patients from cohort 1, those with respiratory diseases, are patients who presented a much higher degree of exposure to toxic substances but also a much higher tendency to smoke, which leads us to appreciate that smoking and exposure to noxes present a potential increased risk for lung function. We have also found that the severity of respiratory dysfunctions can be associated with weight gain, an increase in the number of MS criteria fulfilled, alcohol consumption, active smoking, and a sedentary lifestyle. Excess weight, in turn, is more pronounced in people with a reduced tendency to consume vegetable products and hydrate effectively.

Regarding pulmonary function and respiratory dysfunctions, the comparative analysis of patients with MS and those without MS highlighted an impairment of pulmonary function and a greater severity of respiratory dysfunctions in patients with MS. Moreover, the degree of damage and the severity of respiratory dysfunctions increases with the increase in the number of MS criteria met and the degree of obesity. We could hypothesize that behavioral factors influence pulmonary function through the imbalances caused (MS, obesity, etc.), and MS can be a risk factor for pulmonary function.

Numerous prior research studies have shown a particular interest in exploring the intricate interplay among diverse environmental or behavioral risk factors in direct correlation with their profound implications for pulmonary function, underscoring the critical importance of implementing efficient preventive strategies and targeted interventions in clinical practice.

### 4.2. Involvement of Toxic Exposure and Harmful Habits in the Two Cohorts

In specific workplaces, exposure to pollutants through inhalation, such as dust, fumes, and chemicals, has been identified as a significant contributor to respiratory impairment, speeding up the aging process of the lung. It can be explained by the damage produced to the lung tissues, which further impairs the function of the cells involved in wound healing [43].

Inhaling harmful gases and particles from both tobacco smoking and biomass smoke are widely recognized as environmental risk factors for COPD. However, several analyses have demonstrated that simultaneous exposure to both factors has a synergistic detrimental effect [44,45]. Despite extensive attempts to decrease its prevalence, cigarette smoking stands as the primary cause of premature mortality on a global scale [46,47].

Research conducted in Korea in 2013 involving 1740 male patients sheds light on the critical importance of smoking cessation in individuals diagnosed with COPD. The study concluded that stopping smoking within two years following a COPD diagnosis was linked to a decreased risk of both all-cause mortality and cardiovascular-related deaths compared to individuals who continued smoking, underscoring the profound impact of smoking cessation on improving health outcomes in the population [48].

### 4.3. Intervention on Modifiable Risk Factors of MS in Asthma or COPD Patients

Another emerging significant risk factor for respiratory diseases is obesity, which is associated with decreased lung function and more severe respiratory symptomatology [49,50]. In addition to the effects of excess weight on respiratory mechanics, obesity is a risk factor for bronchial hyper-reactivity [51]. Research indicates that implementing targeted weight loss interventions can lead to significant improvements in asthma symptoms and control among overweight or obese adults facing this condition [52]. Another highly relevant scientific issue is the impact of diabetes mellitus on pulmonary diseases. Studies have confirmed that individuals with diabetes are at a heightened risk of developing several pulmonary conditions, including asthma, COPD, fibrosis, and pneumonia. However, this association does not extend to lung cancer [53].

Moreover, an extensive meta-analysis suggests that about a quarter of asthma patients are also affected by this constellation of metabolic disorders [54]. In the current landscape, there is a notable absence of significant studies targeting the simultaneous reduction of MS components among patients with chronic obstructive bronchial pathology. This aspect underscores a critical area of interest deserving further exploration, with potential implications for future research and intervention.

At the outset, it is remarkable that numerous inquiries have been directed toward evaluating the influence of certain lifestyle factors intricately linked to an escalated risk of MS and their implications on pulmonary function, specifically in COPD or asthmatic patients. Thus, it was stated that regular physical activity enhances disease management and the quality of life for individuals already living with respiratory conditions, suggesting a potential influence of regular exercise on lung function [55,56,57]. Moreover, in a retrospective study involving 101 subjects with COPD, sedentary behavior emerged as an independent predictor of mortality [58]. Over the last few years, there has been growing interest in understanding the role of diet in pulmonary diseases. Research has indicated a link between asthma and dietary habits. Specifically, studies have demonstrated that higher consumption of fast food contributes to increased prevalence of asthma and wheezing [59].

Conversely, a prospective study conducted in the United States found that a diet abundant in fruits, vegetables, and fish might be associated with a lower risk of developing COPD among men [60]. Last but not least, even if exact assessments are challenging, it has been suggested that hydration status plays a significant role in pulmonary function [61]. Research findings indicate a correlation between the consumption of soft drinks and the increased risk of obesity and diabetes. This association is believed to stem from the significant quantities of high fructose corn syrup used in soft drink production, leading to elevated blood glucose levels and potentially dangerous increases in BMI [62]. Moreover, consuming high amounts of red meat, sugary treats, and fried foods is associated with an elevated risk of developing insulin resistance and type 2 diabetes mellitus [63].

The statistical processing of the data from our study highlighted a strong correlation between the intensity of consumption of fast food products and sweet drinks and being overweight in both groups of patients. Diet is an essential factor in the management of metabolic disorders, the analysis of eating habits, and the effective intervention in correcting food excesses and unhealthy habits, which are essential tools for improving chronic pathologies.

Thus, further investigation of diet’s role as a potential modifiable risk factor in COPD remains an area of interest.

Understanding and addressing the interplay between MS and lung dysfunction is crucial for improving patient outcomes and preventing the progression of both metabolic and respiratory diseases. Insulin resistance and hyperglycemia may further deteriorate pulmonary health by affecting the smooth muscle function in the airways [64]. Simultaneously, increased glucose levels in airway fluids create a favorable environment for the proliferation and colonization of bacteria, thereby promoting respiratory infections that can trigger exacerbations of underlying lung diseases [65]. It is essential to intervene to effectively protect people with a high risk of exposure to noxious agents, but also by educating the population to quit smoking and consume foods with protective factors for the body, vegetable products (sources of antioxidants, prebiotics, vitamins, and salt minerals), and food products rich in probiotics, and also to hydrate correctly, helping the optimal detoxification of the body and the good functioning of the intestinal microbiome. Physical activity has a positive impact in stimulating blood and lymphatic circulation and oxygenating the body but also in psycho-emotional balancing. Enhancing patients’ understanding of dietary practices, attitudes, and knowledge is essential for managing chronic conditions effectively and is a crucial component of comprehensive disease care.

### 4.4. Limitations

The present study has several limitations, notably related to the exclusion of patients experiencing exacerbations of their respiratory disease or diabetic ketoacidosis, as well as those with severely altered clinical conditions. This exclusion may have underestimated the severity of respiratory dysfunction and diabetic complications, as exacerbations and decompensation periods often lead to declines in lung volumes and significant changes in biological markers. Furthermore, excluding patients unable to maintain an upright position without support and with conditions impacting anthropological measurements or status-contraindicating spirometry might have restricted the generalizability of the findings.

## 5. Conclusions

The present study shows that metabolic syndrome could be a risk factor for lung function deterioration; moreover, MS is generated by various dietary and lifestyle imbalances that concomitantly occur with aging. Thus, optimizing diet quality to prevent excess weight, avoiding alcohol and tobacco consumption, and reducing respiratory noxious exposure associated with physical activity have a substantial role in maintaining a healthy life.

## Figures and Tables

**Table 1:** Socio-demographic and exposure to various risk factors of patients from cohort 1.

Variable	Categories	Total		M		F		*p*-Value		
		N	%	N	%	N	%	T/M	M/F	T/F
Age group	66–75	25.00	31.25	16.00	31.37	9.00	31.03	0.30	0.22	0.05
	56–65	27.00	33.75	18.00	35.29	9.00	31.03			
	46–55	14.00	17.50	9.00	17.65	5.00	17.24			
	<45	7.00	8.75	6.00	11.76	1.00	3.45			
	>75	7.00	8.75	2.00	3.92	5.00	17.24			
BMI	Normal weight	25.00	31.25	16.00	31.37	9.00	31.03	0.36	0.27	0.09
	Overweight	25.00	31.25	15.00	29.41	10.00	34.48			
	Underweight	3.00	3.75	2.00	3.92	1.00	3.45			
	Obesity I	18.00	22.50	12.00	23.53	6.00	20.69			
	Obesity II	5.00	6.25	3.00	5.88	2.00	6.90			
	Obesity III	4.00	5.00	3.00	5.88	1.00	3.45			
Smoking status	former smoker	29.00	36.25	25.00	49.02	4.00	13.79	0.17	0.37	0.02 *
	active smoker	26.00	32.50	20.00	39.22	6.00	20.69			
	never smoker	25.00	31.25	6.00	11.76	19.00	65.52			
Exposure to respiratory noxious	no	45.00	56.25	25.00	49.02	20.00	68.97	0.10	0.18	0.07
	yes	35.00	43.75	26.00	50.98	9.00	31.03			
MS status	no	27.00	33.75	19.00	37.25	8.00	27.59	0.58	0.24	0.16
MS conditions	MS = 0	3.00	3.75	2.00	3.92	1.00	3.45			
	MS = 1	5.00	6.25	5.00	9.80	0.00	0.00			
	MS = 2	19.00	23.75	12.00	23.53	7.00	24.14			
MS status	yes	53.00	66.25	32.00	62.75	21.00	72.41	0.37	0.44	0.16
MS conditions	MS = 3	15.00	18.75	8.00	15.69	7.00	24.14			
	MS = 4	12.00	15.00	9.00	17.65	3.00	10.34			
	MS = 5	26.00	32.50	15.00	29.41	11.00	37.93			
Residence	Rural	45.00	56.25	30.00	58.82	15.00	51.72	0.16	0.13	0.03 *
	Urban	35.00	43.75	21.00	41.18	14.00	48.28			
Level of education	General/primary education (without a baccalaureate degree)	42.00	52.50	27.00	52.94	15.00	51.72	0.53	0.44	0.23
	Secondary education (baccalaureate diploma)	13.00	16.25	9.00	17.65	4.00	13.79			
	Post-secondary studies	21.00	26.25	13.00	25.49	8.00	27.59			
	Postgraduate studies (master, residency, doctorate, other specialization)	2.00	2.50	1.00	1.96	1.00	3.45			
	Higher education (bachelor’s degree)	2.00	2.50	1.00	1.96	1.00	3.45			
Occupation Status	Retired	4.00	5.00	2.00	3.92	2.00	6.90	0.62	0.54	0.34
	Householder	8.00	10.00	5.00	9.80	3.00	10.34			
	Employed/I go to work every day	14.00	17.50	9.00	17.65	5.00	17.24			
	Socially assisted	53.00	66.25	34.00	66.67	19.00	65.52			
	Unemployed	1.00	1.25	1.00	1.96	0.00	0.00			
Alcohol consumption	No	51	63.75	26	50.98	25	86.20	0.50	0.46	0.31
	Yes, chronic	10	12.50	10	19.60	0	0.00			
	Yes, occasionally	19	23.75	15	29.41	4	13.79			

* *p* < 0.05, statistically significant differences between frequencies; MS = metabolic syndrome; MS 0–5 = the number of criteria met for the MS; N = number (frequency); % = relative frequency, expressed as percent; T = total patients; M = male; F = female.

**Table 2:** Characteristics of patients from cohort 1 stratified by MS score.

MS Score			T		MS-0		MS-1		MS-2		MS-3		MS-4		MS-5	
Frequency/Relative Frequency			N	%	N	%	N	%	N	%	N	%	N	%	N	%
Median Age (years)			62.5		61.3		56.8		56.0		66.4		63.3		66.0	
Gender		F	29.0	36.3	1.0	33.3	0.0	0.0	7.0	36.8	7.0	46.7	3.0	25.0	11.0	42.3
		M	51.0	63.8	2.0	66.7	5.0	100.0	12.0	63.2	8.0	53.3	9.0	75.0	15.0	57.7
Body weight		normal weight	25.0	31.3	3.0	100.0	3.0	60.0	9.0	47.4	8.0	53.3	2.0	16.7	0.0	0.0
		obesity I	18.0	22.5	0.0	0.0	0.0	0.0	4.0	21.1	2.0	13.3	3.0	25.0	9.0	34.6
		obesity II	5.0	6.3	0.0	0.0	0.0	0.0	1.0	5.3	2.0	13.3	0.0	0.0	2.0	7.7
		obesity III	4.0	5.0	0.0	0.0	0.0	0.0	0.0	0.0	0.0	0.0	1.0	8.3	3.0	11.5
		overweight	25.0	31.3	0.0	0.0	1.0	20.0	4.0	21.1	2.0	13.3	6.0	50.0	12.0	46.2
		underweight	3.0	3.8	0.0	0.0	1.0	20.0	1.0	5.3	1.0	6.7	0.0	0.0	0.0	0.0
Spirometry	DVM	mild	1.0	1.3	0.0	0.0	0.0	0.0	1.0	5.3	0.0	0.0	0.0	0.0	0.0	0.0
		moderate	4.0	5.0	0.0	0.0	0.0	0.0	0.0	0.0	2.0	13.3	0.0	0.0	2.0	7.7
		moderate-severe	7.0	8.8	1.0	33.3	0.0	0.0	0.0	0.0	1.0	6.7	2.0	16.7	3.0	11.5
		severe	14.0	17.5	0.0	0.0	0.0	0.0	3.0	15.8	3.0	20.0	3.0	25.0	5.0	19.2
		very severe	19.0	23.8	0.0	0.0	3.0	60.0	7.0	36.8	3.0	20.0	3.0	25.0	3.0	11.5
	DVO	mild	3.0	3.8	0.0	0.0	0.0	0.0	0.0	0.0	0.0	0.0	1.0	8.3	2.0	7.7
		moderate	5.0	6.3	2.0	66.7	1.0	20.0	1.0	5.3	1.0	6.7	0.0	0.0	0.0	0.0
		moderate-severe	1.0	1.3	0.0	0.0	0.0	0.0	1.0	5.3	0.0	0.0	0.0	0.0	0.0	0.0
	DVR	mild	2.0	2.5	0.0	0.0	0.0	0.0	0.0	0.0	1.0	6.7	0.0	0.0	1.0	3.8
		moderate	4.0	5.0	0.0	0.0	0.0	0.0	0.0	0.0	0.0	0.0	1.0	8.3	3.0	11.5
		moderate-severe	2.0	2.5	0.0	0.0	0.0	0.0	0.0	0.0	0.0	0.0	0.0	0.0	2.0	7.7
		severe	2.0	2.5	0.0	0.0	0.0	0.0	0.0	0.0	0.0	0.0	1.0	8.3	1.0	3.8
	Normal		16.0	20.0	0.0	0.0	1.0	20.0	6.0	31.6	4.0	26.7	1.0	8.3	4.0	15.4

MS = metabolic syndrome; MS 0–5 = the number of criteria met for the MS; N = number (frequency); % = relative frequency, expressed as percent; T = total patients; M = male; F = female; DVM = mixed respiratory dysfunction; DVO = obstructive respiratory dysfunction; DVR = restrictive respiratory dysfunction.

**Table 3:** Socio-demographic and exposure to some risk factors of patients from cohort 2.

Variable	Categories	Total		F		M		*p*-Value		
		N	%	N	%	N	%	T/F	F/M	M/T
Age group	36–45	5	6.25	2	4.55	3	8.33	0.25	0.68	0.16
	46–55	21	26.25	9	20.45	12	33.33			
	56–65	30	37.50	16	36.36	14	38.89			
	66–75	21	26.25	14	31.82	7	19.44			
	76–85	3	3.75	3	6.82	0	0			
Total	-	80	-	44	55	36	45			
BMI status	Normal weight	12	15.00	9	20.45	3	8.33	0.22	0.70	0.16
	Obesity I	23	28.75	17	38.64	6	16.67			
	Obesity II	9	11.25	4	9.09	5	13.89			
	Obesity III	5	6.25	3	6.2	2	5.56			
	Overweight	31	38.75	11	25.00	20	55.56			
Smoking status	active smoker	18	22.50	6	13.64	12	33.33	0.30	0.74	0.09
	former smoker	22	27.50	8	18.18	14	38.89			
	never smoker	40	50.00	30	68.18	10	27.78			
Exposure to respiratory noxious	No	65	81.25	38	86.36	27	75.00	0.30	0.74	0.09
	Yes	15	18.75	6	13.64	9	25.00			
MS conditions	MS-3	11	13.75	5	45.45	6	54.55	0.34	0.72	0.20
	MS-4	26	32.50	12	46.15	14	53.85			
	MS-5	43	53.75	27	62.79	16	37.21			
Level of education	General/primary education (without a baccalaureate degree)	39	48.75	22	50.00	17	47.22	0.22	0.70	0.16
	Secondary education (baccalaureate diploma)	8	10.00	5	11.36	3	8.33			
	Post-secondary studies	19	23.75	10	22.72	9	25.00			
	Postgraduate studies (master, residency, doctorate, other specialization)	3	3.75	1	2.27	2	5.56			
	Higher education (bachelor’s degree)	11	13.75	6	13.63	5	13.89			
Occupation status	Retired	50	62.5	32	72.72	18	50.00	0.25	0.68	0.16
	Employed/I go to work every day	14	17.5	6	13.63	8	22.22			
	Householder	12	15.00	4	9.03	8	22.22			
	Socially assisted	3	3.75	1	2.27	2	5.56			
	Unemployed	1	1.25	1	2.27	0	0			
Residence	Rural	34	42.5	19	43.18	15	41,67	0.30	0.74	0.09
	Urban	46	57.5	25	56.81	21	58.33			
Alcohol consumption	No	46	57.5	36	81.81	10	27.78	0.34	0.72	0.20
	Yes, chronic	10	12.5	0	0	10	27.78			
	Yes, occasionally	24	30	8	18.18	16	44.44			

MS = metabolic syndrome; MS 0–5 = the number of criteria met for the MS. N = number (frequency); % = relative frequency, expressed as percent; T = total patients; M = male; F = female.

**Table 4:** Characteristics of patients from cohort 2 stratified by MS score.

MS Score			T		MS-3		MS-4		MS-5	
Frequency/Relative Frequency			N	%	N	%	N	%	N	%
Median Age (years)			60.1		61.5		57.8		61.2	
Gender		F	44.0	55.0	5.0	45.5	12.0	46.2	27.0	62.8
		M	36.0	45.0	6.0	54.5	14.0	53.8	16.0	37.2
Body weight		normal weight	12.0	15.0	3.0	27.3	7.0	26.9	2.0	4.7
		obesity I	23.0	28.8	3.0	27.3	4.0	15.4	16.0	37.2
		obesity II	8.0	10.0	1.0	9.1	3.0	11.5	4.0	9.3
		obesity II	1.0	1.3	0.0	0.0	1.0	3.8	0.0	0.0
		obesity III	5.0	6.3	1.0	9.1	1.0	3.8	3.0	7.0
		overweight	31.0	38.8	3.0	27.3	10.0	38.5	18.0	41.9
Spirometry	DVM	moderate	1.0	1.3	0.0	0.0	0.0	0.0	1.0	2.3
		severe	1.0	1.3	0.0	0.0	1.0	3.8	0.0	0.0
	DVR	mild	4.0	5.0	0.0	0.0	3.0	11.5	1.0	2.3
		moderate	3.0	3.8	1.0	9.1	0.0	0.0	2.0	4.7
	Normal		71.0	88.8	10.0	90.9	22.0	84.6	39.0	90.7

MS = metabolic syndrome; MS 0–5 = the number of criteria met for the MS; N = number (frequency); % = relative frequency, expressed as percent; T = total patients; M = male; F = female; DVM = mixed respiratory dysfunction; DVR = restrictive respiratory dysfunction.

## Data Availability

The original contributions presented in the study are included in the article/Supplementary Material, further inquiries can be directed to the corresponding author.

## Supplementary Materials

The following supporting information can be downloaded at: https://www.mdpi.com/article/10.3390/nu16121851/s1, Tables S1–S8: The Correlation Matrix of principal component analysis; questionnaire for evaluation of behavioral risk factors.
