# Supplementary material for: Integrative Approach to Risk Factors in Simple Chronic Obstructive Airway Diseases of the Lung or Associated with Metabolic Syndrome—Analysis and Prediction
Source: Nutrients. 2024 Jun 13;16(12):1851. doi: 10.3390/nu16121851 (PMC11206714; doi:10.3390/nu16121851)
Supplement: Supplementary file 1 [file nutrients-16-01851-s001.zip › nutrients-3055909-supplementary.pdf]

Supplementary Materials:

Integrative Approach to Risk Factors in Simple Chronic Ob-structive Airway Diseases of the Lung or Associated with Metabolic Syndrome, Analysis and Prediction

Table S1. The Correlation Matrix between MS parameters and FEV1, Pulmonary disease (asthma/COPD), and Diabetes. Legend: No MS = the diagnostic criteria for metabolic syndrome were not met; MS 0-5 = number of metabolic syndrome criteria present: 0-5; BGL = blood glucose level; IFG = impaired fasting glucose; Pulm. Disease = pulmonary disease (asthma/COPD); FEV1 = forced expiratory volume in one second; DM known = patient with a past diagnosis of Diabetes mellitus.

Variables	TGL	BMI	HDL	BGL	FEV	Obesity I-III	Overweight	DM-KNOWN	IFG	Pulm. Disease
TGL	**1**	**0.921**	-0.816	0.867	-0.007	0.675	0.854	0.638	0.644	0.454
BMI	**0.921**	**1**	-0.567	**0.981**	0.294	0.864	**0.939**	0.774	0.855	0.744
HDL	-0.816	-0.567	**1**	-0.536	0.215	-0.374	-0.590	-0.454	-0.246	-0.055
BGL	0.867	**0.981**	-0.536	**1**	0.452	**0.933**	**0.954**	0.845	**0.908**	0.824
FEV	-0.007	0.294	0.215	0.452	**1**	0.606	0.315	0.466	0.716	0.707
Obesity I-III	0.675	0.864	-0.374	**0.933**	0.606	**1**	**0.942**	**0.958**	0.858	**0.946**
Overweight	0.854	**0.939**	-0.590	**0.954**	0.315	**0.942**	**1**	**0.936**	0.761	0.816
DM-KNOWN	0.638	0.774	-0.454	0.845	0.466	**0.958**	**0.936**	**1**	0.679	0.872
IFG	0.644	0.855	-0.246	**0.908**	0.716	0.858	0.761	0.679	**1**	0.837
Pulm. Disease	0.454	0.744	-0.055	0.824	0.707	**0.946**	0.816	0.872	0.837	**1**

Values in bold are different from 0 with a significance level alpha=0.05

Table S2. The Correlation Matrix for the association of various diet habits in different number of metabolic syndrome (MS) criteria present (M0-5). Legend: fast = fast food; sw/pas = sweets/pastries; s-dr = sweet drinks; veg = vegetable products; fru = fruits; wat = water.

DVM	DVO	DVR	Normal	
**0.973**	0.289	0.735	**0.822**	Fast 1
**0.938**	0.338	0.791	0.686	Fast 2
0.053	-0.447	0.060	-0.349	Fast 3
0.418	0.000	-0.331	0.773	Fast 4
0.158	-0.447	-0.120	0.279	sw/pas 1
0.372	0.728	0.065	0.417	sw/pas 2
0.585	0.642	**0.821**	0.444	sw/pas 3
**0.916**	0.133	0.758	0.554	sw/pas 4
0.802	0.058	0.140	**0.897**	sw/pas 5
**0.833**	0.464	0.730	0.780	s-dr.1
0.581	0.149	0.777	0.070	s-dr.2
0.592	0.743	0.682	0.537	s-dr.3
**0.959**	0.000	0.678	0.720	s-dr.4
0.758	-0.267	0.339	0.522	s-dr.5
0.315	0.000	-0.468	0.625	s-dr.6
**1**	0.197	0.647	**0.830**	DVM
0.197	**1**	0.267	0.312	DVO
0.647	0.267	**1**	0.230	DVR
**0.830**	0.312	0.230	**1**	Normal

Values in bold are different from 0 with a significance level alpha=0.05

Table S3. The Correlation Matrix between spirometry pattern (DVM, DVO, DVR. and Normal). healthy diet (vegetable products, fruits, water consumption). and the number of metabolic syndrome (MS) criteria present (M0-5). Fru 1,2, veg 1,2,4, are linked with F2. Most variables are associated with F1. Legend: veg = vegetable products; fru = fruits; wat = water.

DVM	DVO	DVR	Normal	
0.438	-0.218	0.350	0.409	fru 1
0.197	-0.333	0.401	0.156	fru 2
**0.814**	0.276	**0.965**	0.456	fru 3
**0.957**	0.381	0.491	**0.908**	fru 4
0.742	0.494	0.226	**0.826**	fru 5
0.158	-0.447	-0.120	0.279	veg 1
0.663	-0.046	0.595	0.565	veg 2
**0.831**	0.423	**0.853**	0.492	veg 3
0.584	0.391	-0.017	0.773	veg 4
0.475	0.447	0.299	0.349	veg 5
**0.827**	0.333	0.401	**0.937**	wat 1
0.811	0.470	0.704	0.779	wat 2
**0.975**	0.197	0.797	0.709	wat 3
**0.907**	0.530	0.558	0.807	wat 4
-0.774	-0.728	-0.519	-0.720	wat 5
**1**	0.197	0.647	**0.830**	DVM
0.197	**1**	0.267	0.312	DVO
0.647	0.267	**1**	0.230	DVR
**0.830**	0.312	0.230	**1**	Normal

Values in bold are different from 0 with a significance level alpha=0.05

Table S4. The Correlation Matrix between BMI classes (underweight, normal weight, overweight, Obesity I-III), gender (F and M), unhealthy diet (fast food, sweets, pastries, and sweet drinks consumption), and the number of metabolic syndrome (MS) criteria present (M0-5). F1 is associated with almost all parameters. F2 is related to fast 4 and sw/pas 1. normal weight. and underweight. Legend: fast = fast food; sw/pas = sweets/pastries; s-dr = sweet drinks.

Variables	Fast 1	Fast 2	Fast 3	Fast 4	sw/pas 1	sw/pas 2	sw/pas 3	sw/pas 4	sw/pas 5	s-dr.1	s-dr.2	s-dr.3	s-dr.4	s-dr.5	s-dr.6
Normal weight	0.058	-0.148	-0.299	**0.947**	0.530	0.162	-0.387	-0.148	0.672	0.013	-0.530	-0.249	0.088	0.262	0.773
Obesity I	0.645	0.674	0.357	0.564	0.051	0.636	0.104	0.753	**0.813**	0.393	0.459	0.233	0.712	0.792	0.684
Obesity II	**0.845**	0.628	-0.415	0.525	0.581	-0.045	0.579	0.584	0.743	**0.870**	0.249	0.483	**0.812**	0.546	0.000
Obesity III	0.682	0.790	0.135	-0.426	-0.270	0.146	**0.815**	0.748	0.052	0.668	0.809	0.700	0.612	0.282	-0.452
Over weight	**0.826**	**0.933**	0.204	-0.205	-0.241	0.312	0.783	**0.896**	0.296	0.741	**0.835**	0.724	0.771	0.477	-0.166
Under weight	0.026	-0.169	-0.447	0.707	0.447	-0.243	-0.275	-0.310	0.406	0.052	-0.745	-0.186	0.000	0.000	0.667
F	**0.970**	**0.871**	-0.213	0.398	0.252	0.305	0.731	**0.816**	0.746	**0.939**	0.523	0.692	**0.900**	0.614	0.087
M	**0.964**	**0.989**	0.052	0.248	-0.052	0.426	0.697	**0.908**	0.659	**0.850**	0.611	0.717	**0.896**	0.626	0.273

Values in bold are different from 0 with a significance level alpha=0.05

Table S5. The Correlation Matrix between (BMI status. respiratory dysfunctions. gender. and sport/physical exercises) and the number of metabolic syndrome (MS) criteria present (M0-5). F1 is associated with gender, sport practice (1-5). Obesity II-III and overweight, DVM and DVO, M0, M1, M5. F2 is associated with normal weight. normal respiratory status and underweight. Legend: spr = sport (physical activity).

Variables	DVM	DVO	DVR	Normal	Normal weight	Obesity I	Obesity II	Obesity III	Over weight	Under weight
DVM	**1**	0.197	0.647	**0.830**	0.140	0.794	0.768	0.605	0.799	0.039
DVO	0.197	**1**	0.267	0.312	-0.052	0.114	0.186	0.302	0.290	-0.333
DVR	0.647	0.267	**1**	0.230	-0.613	0.168	0.571	**0.987**	**0.937**	-0.535
Normal	**0.830**	0.312	0.230	**1**	0.587	0.730	0.754	0.165	0.395	0.469
Normal weight	0.140	-0.052	-0.613	0.587	**1**	0.428	0.239	-0.683	-0.476	0.773
Obesity I	0.794	0.114	0.168	0.730	0.428	**1**	0.360	0.172	0.459	0.114
Obesity II	0.768	0.186	0.571	0.754	0.239	0.360	**1**	0.448	0.516	0.186
Obesity III	0.605	0.302	**0.987**	0.165	-0.683	0.172	0.448	**1**	**0.951**	-0.603
Over weight	0.799	0.290	**0.937**	0.395	-0.476	0.459	0.516	**0.951**	**1**	-0.456
Under weight	0.039	-0.333	-0.535	0.469	0.773	0.114	0.186	-0.603	-0.456	**1**
F	**0.926**	0.390	0.724	0.805	0.096	0.587	**0.909**	0.653	0.767	-0.043
M	**0.972**	0.273	0.719	0.768	-0.018	0.734	0.674	0.706	**0.879**	-0.039
spr 1	**0.917**	0.243	0.454	**0.928**	0.425	0.719	**0.901**	0.366	0.553	0.243
spr 2	0.752	0.707	0.520	0.773	0.073	0.564	0.525	0.533	0.675	0.000
spr 3	0.591	-0.333	0.668	0.156	-0.258	0.342	0.557	0.603	0.622	-0.333
spr 4	0.748	0.655	0.787	0.613	-0.236	0.373	0.608	0.790	**0.842**	-0.218
spr 5	**0.992**	0.248	0.734	0.791	0.035	0.723	0.783	0.694	**0.857**	-0.035

Values in bold are different from 0 with a significance level alpha=0.05

Table S6. The Correlation Matrix of principal component analysis for age, HDL, HbA1c. TGL, MS Cond and diet (water, fruits, fish, alcohol, vegetable, bread), Age, HbA1c, and almost all diet aspects are linked with PC2, while TGL and HDL are associated with PC1. Legend: fast = fast food; sw/pas = sweets/pastries; s-dr = sweet drinks; veg = vegetable products; MS Cond = Metabolic syndrome conditions met (MS 3-5); HbA1c = glycosylated hemoglobin.

Variables	Age	HbA1c	TGL	HDL	MS Cond	Fast	Veg	Fruits	s-dr.	Alcohol	Fish	sw-ps	Bread	Water	Sport
Age	**1**	-0.217	-0.016	0.059	0.056	**-0.342**	**-0.238**	0.064	**-0.273**	-0.063	0.086	-0.189	-0.178	**0.296**	0.041
HbA1c	-0.217	**1**	-0.033	-0.018	-0.041	**0.324**	0.199	-0.039	**0.410**	0.085	**0.231**	0.204	**0.257**	-0.202	-0.086
TGL	-0.016	-0.033	**1**	**-0.419**	**0.297**	-0.112	0.170	-0.035	0.001	-0.020	-0.086	0.059	0.157	-0.092	0.025
HDL	0.059	-0.018	**-0.419**	**1**	-0.184	-0.075	**-0.290**	-0.011	-0.085	0.044	-0.122	-0.153	-0.094	-0.041	-0.179
MS Cond	0.056	-0.041	**0.297**	-0.184	**1**	-0.105	0.130	-0.031	-0.082	-0.098	-0.034	0.006	0.091	-0.101	0.191
Fast	**-0.342**	**0.324**	-0.112	-0.075	-0.105	**1**	0.192	-0.093	**0.393**	-0.027	0.018	**0.372**	**0.272**	-0.041	0.043
Veg	**-0.238**	0.199	0.170	**-0.290**	0.130	0.192	**1**	**0.256**	**0.305**	0.117	0.173	**0.237**	**0.266**	-0.059	-0.003
Fruits	0.064	-0.039	-0.035	-0.011	-0.031	-0.093	**0.256**	**1**	-0.004	-0.041	0.112	-0.003	0.196	**0.237**	-0.018
s-dr.	**-0.273**	**0.410**	0.001	-0.085	-0.082	**0.393**	**0.305**	-0.004	**1**	**0.326**	0.195	**0.282**	**0.312**	-0.077	0.027
Alcohol	-0.063	0.085	-0.020	0.044	-0.098	-0.027	0.117	-0.041	**0.326**	**1**	0.150	0.089	0.104	-0.004	0.040
Fish	0.086	**0.231**	-0.086	-0.122	-0.034	0.018	0.173	0.112	0.195	0.150	**1**	**0.231**	0.084	0.169	0.046

sw-ps	-0.189	0.204	0.059	-0.153	0.006	**0.372**	**0.237**	-0.003	**0.282**	0.089	**0.231**	**1**	**0.326**	0.107	-0.017
Bread	-0.178	**0.257**	0.157	-0.094	0.091	**0.272**	**0.266**	0.196	**0.312**	0.104	0.084	**0.326**	**1**	-0.003	-0.026
Water	**0.296**	-0.202	-0.092	-0.041	-0.101	-0.041	-0.059	**0.237**	-0.077	-0.004	0.169	0.107	-0.003	**1**	-0.030
Sport	0.041	-0.086	0.025	-0.179	0.191	0.043	-0.003	-0.018	0.027	0.040	0.046	-0.017	-0.026	-0.030	**1**

Values in bold are different from 0 with a significance level alpha=0.05

Table S7. The Correlation Matrix between alcohol and tobacco consumption and BMI status and age in various MS diabetes patients. Legend: Metabolic syndrome conditions met (MS 3-5); alc = alcohol consumption (1-6).

Variables	Alc-1	Alc-2	Alc-3	Alc-4	Alc-5	Alc-6	active smoker	former smoker	never smoker	Normal weight	Obesity I	Obesity II	Obesity III	Over weight	36-45	46-55	56-65	66-75
Alc-1	**1**	0.908	0.908	0.693	0.817	0.908	0.996	0.995	0.985	-0.363	0.908	0.817	0.583	0.976	0.091	0.767	0.996	0.908
Alc-2	0.908	**1**	**1.000**	0.327	0.500	**1.000**	0.866	0.945	0.822	-0.721	**1.000**	0.500	0.189	0.977	0.500	0.427	0.866	**1.000**
Alc-3	0.908	**1.000**	**1**	0.327	0.500	**1.000**	0.866	0.945	0.822	-0.721	**1.000**	0.500	0.189	0.977	0.500	0.427	0.866	**1.000**
Alc-4	0.693	0.327	0.327	**1**	0.982	0.327	0.756	0.619	0.807	0.419	0.327	0.982	0.990	0.520	-0.655	0.994	0.756	0.327
Alc-5	0.817	0.500	0.500	0.982	**1**	0.500	0.866	0.756	0.904	0.240	0.500	**1.000**	0.945	0.672	-0.500	0.997	0.866	0.500
Alc-6	0.908	**1.000**	**1.000**	0.327	0.500	**1**	0.866	0.945	0.822	-0.721	**1.000**	0.500	0.189	0.977	0.500	0.427	0.866	**1.000**
active smoker	0.996	0.866	0.866	0.756	0.866	0.866	**1**	0.982	0.997	-0.277	0.866	0.866	0.655	0.952	0.000	0.822	**1.000**	0.866
former smoker	0.995	0.945	0.945	0.619	0.756	0.945	0.982	**1**	0.963	-0.454	0.945	0.756	0.500	0.993	0.189	0.700	0.982	0.945
never smoker	0.985	0.822	0.822	0.807	0.904	0.822	0.997	0.963	**1**	-0.197	0.822	0.904	0.715	0.924	-0.082	0.866	0.997	0.822
Normal weight	-0.363	-0.721	-0.721	0.419	0.240	-0.721	-0.277	-0.454	-0.197	**1**	-0.721	0.240	0.545	-0.558	-0.961	0.319	-0.277	-0.721
Obesity I	0.908	**1.000**	**1.000**	0.327	0.500	**1.000**	0.866	0.945	0.822	-0.721	**1**	0.500	0.189	0.977	0.500	0.427	0.866	**1.000**
Obesity II	0.817	0.500	0.500	0.982	**1.000**	0.500	0.866	0.756	0.904	0.240	0.500	**1**	0.945	0.672	-0.500	0.997	0.866	0.500
Obesity III	0.583	0.189	0.189	0.990	0.945	0.189	0.655	0.500	0.715	0.545	0.189	0.945	**1**	0.392	-0.756	0.969	0.655	0.189
Over weight	0.976	0.977	0.977	0.520	0.672	0.977	0.952	0.993	0.924	-0.558	0.977	0.672	0.392	**1**	0.305	0.609	0.952	0.977
36-45	0.091	0.500	0.500	-0.655	-0.500	0.500	0.000	0.189	-0.082	-0.961	0.500	-0.500	-0.756	0.305	**1**	-0.569	0.000	0.500
46-55	0.767	0.427	0.427	0.994	0.997	0.427	0.822	0.700	0.866	0.319	0.427	0.997	0.969	0.609	-0.569	**1**	0.822	0.427
56-65	0.996	0.866	0.866	0.756	0.866	0.866	**1.000**	0.982	0.997	-0.277	0.866	0.866	0.655	0.952	0.000	0.822	**1**	0.866
66-75	0.908	**1.000**	**1.000**	0.327	0.500	**1.000**	0.866	0.945	0.822	-0.721	**1.000**	0.500	0.189	0.977	0.500	0.427	0.866	**1**

Values in bold are different from 0 with a significance level alpha=0.05

Table S8. The Correlation Matrix between diet and BMI status in all MS 3,4,5.

Variables	Normal weight	Obesity I	Obesity II	Obesity III	Over weight
Fast-1	-0.301	0.878	0.853	0.635	0.960
Fast-2	0.127	0.596	0.993	0.901	0.752
Fast-3	-0.817	0.189	-0.756	-0.929	-0.023
Fast-4	-0.721	**1.000**	0.500	0.189	0.977
s-dr.1	0.327	0.419	0.996	0.971	0.602
s-dr.2	-0.558	0.977	0.672	0.392	**1.000**
s-dr.3	-0.721	**1.000**	0.500	0.189	0.977
s-dr.4	-0.721	**1.000**	0.500	0.189	0.977

s-dr.5	0.817	-0.189	0.756	0.929	0.023
sw/pas-1	0.127	0.596	0.993	0.901	0.752
sw/pas-2	-0.038	0.721	0.961	0.817	0.851
sw/pas-3	-0.781	0.996	0.419	0.099	0.954
sw/pas-4	-0.091	0.756	0.945	0.786	0.877
sw/pas-5	-0.386	0.918	0.803	0.564	0.981
Bread-2	0.350	0.397	0.993	0.976	0.583
Bread-3	-0.687	**0.999**	0.540	0.235	0.986
Bread-4	0.240	0.500	**1.000**	0.945	0.672
Bread-5	0.240	0.500	**1.000**	0.945	0.672
Normal weight	**1**	-0.721	0.240	0.545	-0.558
Obesity I	-0.721	**1**	0.500	0.189	0.977
Obesity II	0.240	0.500	**1**	0.945	0.672
Obesity III	0.545	0.189	0.945	**1**	0.392
Over weight	-0.558	0.977	0.672	0.392	**1**

Values in bold are different from 0 with a significance level alpha=0.05

Questionnaire for evaluation of behavioral risk factors

Personal data

1. Please mention your age (in years):

2. Please mention your sex:

- ☐ Masculine
- ☐ Feminine
- ☐ Others

3. Please indicate the geographical region where you live?

- ☐ Banat
- ☐ Bukovina
- ☐ Crişana
- ☐ Dobrogea
- ☐ Maramureş County
- ☐ Moldova
- ☐ Muntenia
- ☐ Oltenia
- ☐ Transylvania

4. Please indicate where you currently reside:

- ☐ Town
- ☐ Commune/Village

5. Please mention the level of education:

- ☐ General/primary education (without a baccalaureate degree)

- Secondary education (baccalaureate diploma)
- Post-secondary studies
- Higher education (bachelor's degree)
- Postgraduate studies (master, residency, doctorate, other specializations)

6. Please mention the occupation status:

- Unemployed
- Socially assisted
- Housewife
- Pensioner
- Student/student
- Telework
- I travel to work every day
- I work in a mixed regime (teleworking and going to work)

Anthropometric data

7. Please mention your weight (in kg):

8. Please mention your height (in cm):

9. Which of the following foods do you eat frequently? (multiple variants)

- Hamburgers
- Hot dog
- French fries
- Shaorma
- Packed sandwiches
- Chips
- Snacks
- Patisserie products, pastry
- Packaged cakes
- Candy
- Ice cream
- Other packaged sweet products
- Chewing gum
- Sweetened carbonated drinks
- Sweetened non-carbonated drinks
- Energy drinks
- Coffee
- I do not frequently consume any of the products

10. Which of the following foods do you consume the rarest? (multiple variants)

- Hamburgers
- Hot dog
- French fries
- Shaorma
- Packed sandwiches
- Chips

- Snacks
- Patisserie products, pastry
- Packaged cakes
- Candy
- Ice cream
- Other packaged sweet products
- Chewing gum
- Sweetened carbonated drinks
- Sweetened non-carbonated drinks
- Energy drinks
- Coffee
- None between products

11. Which of the following alimentation products have you never consumed? (multiple variants)

- Hamburgers
- Hot dog
- French fries
- Shaorma
- Packed sandwiches
- Chips
- Snacksuri
- Patisserie products, pastry
- Packaged cakes
- Candy
- Ice cream
- Other packaged sweet products
- Chewing gum
- Sweetened carbonated drinks
- Sweetened non-carbonated drinks
- Energy drinks
- Coffee
- None of the products

12. How often do you eat fast food or ready-to-eat packaged food? (only one option)

- Daily
- 2-3 times a week
- Once a week
- 2-3 times a month
- Very rarely or not at all

13. What is the reason you eat fast food or ready-to-eat packaged food? (several variants)

- Lack of time
- Consumer pleasure
- Convenience
- The temptation caused by the consumption of those around you
- Advertisements
- Boredom
- Satisfying the craving for sweet

- The need to consume something when I work or watch TV
- Others
- I am not used to consuming such products

14. How many servings of vegetables (approx. 100 g) do you consume every day? (single variant)

- Very rarely or not at all
- One
- Two
- Three
- More than three

15. How many servings of fruit (approx. 100 g) do you consume every day? (single variant)

- Very rarely or not at all
- One
- Two
- Three
- More than three

16. How often do you eat meat? (single variant)

- Very rarely or not at all
- 2-3 times a month
- Once a week
- 2-3 times a week
- Daily

17. How often do you consume carbonated or sweetened drinks (1 serving = 330 mL, one glass)? (single variant)

- Very rarely or not at all
- 2-3 times a month
- Once a week
- 2-3 times a week
- Daily more than one serving
- Daily one serving

18. How often do you consume alcoholic beverages (1 glass of wine = 125mL, 1 glass of drink *pure alcohol =50mL)? (single variant)

- Very rarely or not at all
- 2-3 times a month
- Once a week
- 2-3 times a week
- Daily more than one serving
- Daily one serving

19. How often do you consume fish or seafood? (single variant)

- Very rarely or not at all
- 2-3 times a month
- Once a week
- 2-3 times a week
- Daily

20. How often do you consume sweets / pastries? (single variant)

- Very rarely or not at all
- 2-3 times a month
- Once a week
- 2-3 times a week
- Daily

21. How often do you consume pasta, rice or other cereals? (single variant)

- Very rarely or not at all
- 2-3 times a month
- Once a week
- 2-3 times a week
- Daily

22. How often do you consume dairy products? (single variant)

- Very rarely or not at all
- 2-3 times a month
- Once a week
- 2-3 times a week
- Daily

23. How many eggs do you eat per week? (single variant)

- Very rarely or not at all
- 1 - 2 eggs
- 3 - 4 eggs
- 5 - 7 eggs
- More than 7 eggs

24. What category of food do you consume most often? (single variant)

- Fast food products
- Pizza, snacks, pastries, sweets
- Products made from sausages and preserves
- Food cooked in restaurants
- Home-cooked food

25. What type of cooked foods do you eat most often? (single variant)

- Fried foods
- Food prepared at the henhouse on wood or coal
- Grilled food
- Food prepared in the oven
- Boiled or steamed foods
- Thermally unprocessed food
- Food cooked under vacuum
- Others

26. How much water do you drink per day? (single variant)

- Less than 1 L
- 1 l
- 2 l
- 3 l
- Over 3 L

27. What category of liquids are you used to consuming most often? (single variant)

- Alcoholic beverages: sparkling drinks / wine, beer, etc.
- Carbonated or sweetened soft drinks including tonic ones
- Coffee
- Tea
- Natural juices
- Drinking water (from tap, well)
- Still mineral water
- Carbonated mineral water
- Other type of water

28. What category of food predominates in the daily diet? (single variant)

- Vegetables and fruits
- Cereals and pasta
- Dairy
- Fish and seafood dishes
- Meat
- Eggs
- Prepaired from meat (sausages, minced meats, canned food, etc.)
- Pizza, pitchers and pastries
- High-fat foods
- Fast food products

29. How much bread do you consume per day? (single variant)

- More than 12 slices
- 8-12 slices
- 5-7 slices
- 4 slices
- Very rarely or not at all

30. Did you work in a toxic environment or were you exposed to respiratory noxes for various reasons? (single variant)

- Not
- Yes

31. Do you smoke? (single variant)

- Yes, excessive daily
- Yes, 1-2 cigarettes daily
- Yes, 2-3 times a week
- Yes, occasionally
- Not

32. How often do you consume coffee? (one variant)

- Daily
- 2-3 times a week
- Once a week
- 2-3 times a month
- Very rarely or not at all

33. How much coffee do you consume daily? (one variant)

- A cup
- 2-3 cups
- 4-5 cups
- More than 5 cups
- Rarely or not at all consumption

34. Do you usually do sports / exercise? (single variant)

- Not
- Yes, very rarely
- Yes, 2-3 times a week
- Yes, every day under an hour
- Yes, daily for at least an hour
